# Mapping molecular pathways for embryonic Sertoli cells derivation based on differentiation model of mouse embryonic stem cells

**DOI:** 10.1186/s13287-020-01600-2

**Published:** 2020-02-26

**Authors:** Chenze Xu, Yichen Dai, Ali Mohsin, Haifeng Hang, Yingping Zhuang, Meijin Guo

**Affiliations:** 1 State Key Laboratory of Bioreactor Engineering, East China University of Science and Technology, Shanghai, 200237 People’s Republic of China; 2 Engineering Research Centre of Processes System, Ministry of Education, East China University of Science and Technology, 130 Meilong Rd., Shanghai, 200237 China; 3 Institute for Stem Cell and Regeneration, Chinese Academy of Sciences, Beijing, 100101 China

**Keywords:** Embryonic stem cells, Embryonic Sertoli cells, Light-switchable transgene system, Male determination

## Abstract

**Background:**

Embryonic Sertoli cells (eSCs) have been known for playing important roles in male reproductive development system. In current studies, eSCs were mainly generated from induced intermediate mesoderm. The deriving mechanism of eSCs has been unclear so far. Therefore, this work was aimed to reveal the molecular pathways during derivation of eSCs.

**Methods:**

In this scenario, a differentiation model from mouse embryonic stem cells (mESCs) to eSCs was established through spatiotemporal control of 5 key factors, Wilms tumor 1 homolog (*Wt1*), GATA binding protein 4 (*Gata4*), nuclear receptor subfamily 5, group A, member 1 (*Nr5a1*, i.e., *Sf1*), SRY (sex determining region Y)-box 9 (*Sox9*), doublesex, and mab-3 related transcription factor 1 (*Dmrt1*). To investigate the molecular mechanism, these key factors were respectively manipulated through a light-switchable (light-on) system, tetracycline-switchable (Tet-on) system, and CRISPR/Cas9 knock out (KO) system.

**Results:**

Via the established approach, some embryonic Sertoli-like cells (eSLCs) were induced from mESCs and formed ring-like or tubular-like structures. The key factors were respectively manipulated and revealed their roles in the derivation of these eSLCs. Based on these results, some molecular pathways were mapped during the development of coelomic epithelial somatic cells to eSCs.

**Conclusions:**

This differentiation model provided a high controllability of some key factors and brought a novel insight into the deriving mechanism of Sertoli cells.

**Supplementary information:**

accompanies this paper at 10.1186/s13287-020-01600-2.

## Background

In mammalian embryo, Sertoli cells play a key role in the onset of male determination and gonadal development [1]. The mechanism of derivation and development of Sertoli cells has a close relevance with some reproductive disorders [2–5]. However, the molecular pathways in the derivation of Sertoli cells in embryos are still unclear. The most widely accepted theory indicated that the Sertoli cells were mainly derived from coelomic epithelium from mesoderm [6]. Some coelomic epithelial somatic cells went through epithelial-mesenchymal transition (EMT) and developed into nuclear receptor subfamily 5, group A, member 1 (NR5A1, i.e., SF1)-positive cells, as the main precursor cells of Sertoli cells. In this phase, some factors were involved including Wilms tumor 1 homolog (*Wt1*), GATA binding protein 4 (*Gata4*), LIM homeobox protein 9 (*Lhx9*), empty spircles homeobox 2 (*Emx2*), transcription factor 21 (*Pod1*), tripartite motifcontaining 28 (*Tif1β*), nuclear receptor coactivator 2 (*Tif2*), insulin receptor (*Insr*), chromobox 2 (*Cbx2*), sine oculis-related homeobox 1/4 (*Six1/4*), zinc finger protein multitype 2 (*Fog2*), mitogen-activated protein kinase 4 (*Map3k4*), growth arrest, and DNA-damage-inducible 45 gamma (*Gadd45g*) [6–15]. In mice, these SF1-positive precursor cells (SPCs) ingressed into bipotential gonads and became SF1-positive gonadal precursor cells (SGPCs, i.e., pre-Sertoli cells) in 11.2–11.4 day post coitum (*dpc*). SGPCs were the common precursor cells of male gonad supporting cells (Sertoli cells) and female gonadal supporting cells (follicular cells, i.e., granulosa) [6, 16]. After that, these SGPCs developed into embryonic Sertoli cell (eSCs) under the influence of factors including *Wt1*, *Gata4*, *Sry*, SRY-box 9 (*Sox9*), SRY-box 8 (*Sox8*), fibroblast growth factor 9 (*Fgf9*), fibroblast growth factor receptor 2 (*Fgfr2*), prostaglandin D2 (PGD2), prostaglandin D2 synthase (*Ptgds*), anti-Mullerian hormone (*Amh*), doublesex and mab-3 related transcription factor 1 (*Dmrt1*), and glial cell line derived neurotrophic factor (*Gdnf*) [16–21]. Many theories on the deriving mechanism of Sertoli cells have already been proposed; however, still further investigation is needed to explore the complete mechanism of deriving eSCs [1, 6, 16, 22].

The main complications of exploring the deriving mechanism of Sertoli cells are as follows: the cells have a great variety in genital ridges and coelomic epithelium, the relevant factors have complicated interaction, the target genes are hard to manipulate in vivo environment, and so on [6, 14, 18, 22, 23]. Consequently, recently, some studies provided molecular mechanism through inducing Sertoli-like cells from embryonic stem cells by retiotic acid (RA) treatment, reducing the size of cultured ESC colonies, and some other factors [24, 25]. These approaches helped to provide evidences for verifying those established theories. However, there are still some barriers to efficiently produce Sertoli cells [26–30]. Thus, in our former work, we have induced embryonic Sertoli-like cells (eSLCs) from mouse embryonic stem cells (mESCs) by overexpression of *Wt1*, *Gata4*, *Sf1*, *Sry*, *Sox9*, and *Dmrt1* [31]. However, the molecular mechanism was not involved. Therefore, this work established a novel procedure to induce mESCs into eSLCs via controllable regulation of the key factors to create a differentiation model for investigation of the molecular pathways.

In this approach, the cells were manipulated refer to the developmental schedule in vivo. In 0.5–8.5 days, the mESCs were treated by RA and Activin A to induce intermediate mesoderm (IM) [25, 32]. In 9.5–10.5 days, *Wt1*, *Gata4*, and *Sf1* were upregulated through light-switchable (light-on) transgene system [33–36]. In 11.5–12.5 days, *Sox9* and *Dmrt1* were expressed through tetracycline-on (Tet-on) transgene system. At 13.5 days, culture medium was supplemented with recombinant proteins of epidermal growth factor (EGF), PGD2, and FGF9 [37–40]. Results showed a differentiation process from mESCs to eSLCs was established mimetic to the presumptive developmental process in embryos. Furthermore, the induced eSLCs had similar characteristic and expression of specific markers with eSCs including, AMH^+^, FSHR^+^, GDNF^+^, FASL^+^, and EMX2^−^ [1, 41, 42]. Moreover, through the inducing approach, there were ring-like structures and tubular-like structures formed as the same behavior as those eSCs in embryos [6, 43]. Therefore, this approach provides a differentiation model of deriving eSCs from mESCs.

Conclusively, we mapped the molecular mechanism from IM to eSCs based on a differentiation model from mESCs to eSCs. Moreover, this approach will definitely serve in future as a base for further fundamental researches on mechanism studies.

## Methods

### Preparation of lentivirus

Tet-on lentiviral plasmids of *Sox9* and *Dmrt1* were purchased from Addgene (USA) (Additional file 1: Table S1). Sequences of *Wt1*, *Gata4*, and *Sf1* were cloned from cDNA reverse transcription products of mRNA from embryos and testicular extract, and then selectively amplified by PCR. Primers were listed (Additional file 2: Table S2). These sequences were connected to lightOn element (Additional file 7: Figure S1). They were inserted into Addgene plasmid FUW-TetON-GFP by replacing the tetracycline response element via restriction enzyme cutting site *Pst*I and *Bsm*BI. These plasmids with enhanced green fluorescent protein (EGFP) were used to detect the lentiviral infection efficiency. For the experimental use, these target gene sequences were inserted in the FUW-TetON-GFP vector by replacing tetracycline response element and EGFP via site *Pst*I and *Bsr*GI. Then, three plasmids, FUW-LightO-*Wt1*, FUW-LightO-*Gata4*, and FUW-LightO-*Sf1*, were constituted. The constructed plasmids were amplified in *DH5α E. coli* and later extracted by an EndoFree Mini Plasmid Kit II (TIANGEN, China). The light-on system was designed by the researchers in lab of technology creators of the light-switchable transgene expression system (Synthetic Biology and Biotechnology Laboratory, State Key Laboratory of Bioreactor Engineering, Shanghai, Collaborative Innovation Center for Biomanufacturing Technology, East China University of Science and Technology) [33–36].

HEK293T cells were cultured in Opti-MEM (Gibco, USA). Following the manufacturer’s instructions, each group of HEK293T cells was separately transfected with the 5 plasmids (FUW-lightO-*Wt1*, FUW-lightO-*Gata4*, FUW-lightO-*Sf1*, FUW-TetO-*Sox9*, or FUW-TetO-*Dmrt1*) and co-transfected with plasmid psPAX2 and PMD.2G by Lipofectamine3000 (Thermo, USA) (Additional file 4: Table S4). To obtain lentivirus of constitutive light-switchable transactivation factor GAVPO, FUW-lightO-GAVPO was transduced into HEK293T by Lipofectamine3000 (Thermo, USA) (Additional file 7: Figure S1). The supernatant was collected after 48–72 h of post-transfection and was concentrated with a Lenti-Pac™ Lentivirus Concentration Solution (GeneCopoeia, USA), followed by its storage − 80 °C for later use.

In Tet-on system, the cells were infected by the corresponding lentivirus. The target genes expressed under influence of 1–2 μg/ml of doxycycline (DOX) (Sigma, USA), which is a replacement of tetracycline. In light-on system, the cells were infected by the corresponding lentivirus with GAVPO lentivirus. The target genes were activated by being illuminated with 0.84 W/m^2^ blue light using a LED lamp (460 nm peak). Through qPCR, the transcriptional expression of the 5 target genes were detected (Fig. 2k). To detect the transduction efficiency of these systems, the sequence of EGFP was inserted in the plasmids right after the target gene sequence. In a single lentiviral infection, the transduction positive rate was in 50–70% in mESCs.

The genetic KO was performed with corresponding CRISPR/Cas9 KO plasmids by Lipofectamine3000 (Thermo, USA) (Additional file 1: Table S1).

In group (*W.G.S1.S9.D*(constant)) and (*W.G.S1.D*(constant)), the lentiviral plasmid FUW-TetO-*Dmrt1* was replaced by constructed plasmid pLenti-CMV-*Dmrt1* (Additional file 7: Figure S1).

### mESCs line and culture

The mESC used in the current study were derived from R1/E cell line (male gender, 129X1 × 129S1). Mouse embryonic fibroblasts (MEFs) were derived from Kunming white mice between 12.5 and 13.5 *dpc*. Both cell lines were obtained from Chinese Academy of Sciences cell bank (Shanghai, China).

To culture mESCs, MEFs (passage 3, P3) treated with mitomycin C (10 μg/mL, 2–3 h) were seeded in 0.1% gelatin-coated T-flasks as feeder layers. After 12–24 h, mESCs were recovered from nitrogen cryopreservation using mESCs culture medium composed of Dulbecco’s modified Eagle’s medium (DMEM) with 12.5% fetal calf serum (FBS); 0.11 g/L sodium pyruvate; 0.30 g/L l-glutamine; 1.5 g/L sodium bicarbonate; 0.5 g/L HEPES (Gibco, USA); 50.0 μmol β-mercaptoethanol; 1× non-essential amino acids (NEAA); and 1 μg/mL (> 10^3^ U/mL) leukemia inhibitory factor (LIF) (Invitrogen, USA). Culture medium was replaced every day. The cell passage was performed when the cell confluence reached 80%.

In differentiation experiments, IM-inducing medium, basic culture medium, and long-term culture medium were respectively applicated in 0.5–8.5 days, in 8.5–13.5 days, and from 13.5 days. IM-inducing medium was supplemented with 100 mM of RA and 10 ng/mL of Activin A [32]. Basic culture medium was based on the mESC culture medium without LIF and β-mercaptoethanol. The long-term culture medium was based on the mESC culture medium supplemented with 10 ng/mL EGF (Gibco, USA), 10 ng/mL FGF9 (Peprotech, USA) and 1 μg/mL PGD2 (BioGems, USA) [44, 45]. In the three medium, LIF and β-mercaptoethanol were removed. Medium was replaced every 2 days. Cell passages were performed when cell confluence reaches over 80%, and cell dissociation was conducted using collagenase I (Gibco, USA) and 0.1% trypsin-EDTA. The cell passage was performed when the cell confluence reached 90%.

### qPCR (quantitative RT-PCR)

Total RNA from test groups was isolated using Invitrogen™ TRIzol™ (Thermo, USA), then reverse-transcribed by a PrimeScript™ RT reagent Kit with gDNA Eraser (Perfect Real Time) (TAKARA, Japan). qPCR was performed with SYBR Premix Ex Taq™ II (Tli RNaseH Plus) (TAKARA, Japan) according to the manufacturer’s instructions on a CFX96 touch qPCR system (Bio-Rad, USA). Primer design is listed in supplementary material (Additional file 3: Table S3).

### Immunofluorescence (IF)

The cell samples being fixed with 4.0% methanol (10–30 min) were perforated on membrane by Triton X100 (0.1%, 10 min for surface markers, 20 min for cytoplasmic factors, over 30 min for intranuclear factors) and were washed with PBS for three times (10 min per wash). Later they were blocked with 5% bovine serum albumin (BSA) for 30 min and were incubated with antibodies and DAPI (Sigma, USA) according to the manufacturer’s instruction. Followed by washing with PBS as above was incubated with secondary antibodies before being completely ready for observation under an EVOS FL Auto imaging system (Life Technologies, USA). The result of IF-positive cells was acquired at 20 views and counted manually. The antibodies used in this work were listed in supplementary material (Additional file 5: Table S5).

### Flow cytometry (FCM) analysis

Cell samples were dissociated by 0.25% trypsin-EDTA, fixed with 4.0% methanol (10–30 min) and washed with PBS followed by their perforation on membrane by Triton X100 (0.1–0.2%, 10 min for surface markers, 20 min for cytoplasmic factors, over 30 min for intranuclear factors), later were washed again with PBS and were quantified. Then samples were re-suspended in a 100 μL volume of DMEM medium in a concentration of 1 × 10^6^–10^7^ cells/mL. Matched controls of antibody for FCM were applied according to the manufacturer’s instructions using a FACSArial system (BD Biosciences, USA). The quad was set according to parallel samples treated by corresponding isotype antibodies. Antibodies were listed in supplementary material (Additional file 6: Table S6).

### Statistical analysis

Every test groups had at least three parallel samples. In qPCR, results were the average mean of three to four tests for each sample. To detect IF-positive cells, three parallel samples were observed of 20 views respectively, counted manually, and converted into percentage. In experiments inducing eSLCs from mES cells, the experiments were successively repeated three times. In factor analysis, the “+” and “−” results were determined in three parallel samples through IF with specific antibodies. In wound healing assay, the scratch was performed by tips. The result was determined in the next day. Every test groups had three parallel samples. Error bars represent ± SD (standard deviation). Reliable data meet the condition SD / mean < 10%. Experimental data were reported as mean ± SD. Heatmap was expressed as mean value (*n* = 3).

Asterisks indicated statistical significance which was evaluated by one-way ANOVA (analysis of variance) with SPSS software. *P* values < 0.05 were considered statistically significant (*); *P* value < 0.01 had great significant statistical difference (**); *P* value < 0.001 had extreme great significant statistical difference (***).

## Results

### Determination of established differentiation model from mESCs to eSCs

In order to map the molecular differentiation pathways for deriving eSCs, this work aimed to establish a differentiation from mESCs to eSCs to reproduce the expression schedule of key factors and analyze cellular morphology and organic forms.

**Fig. 1:**
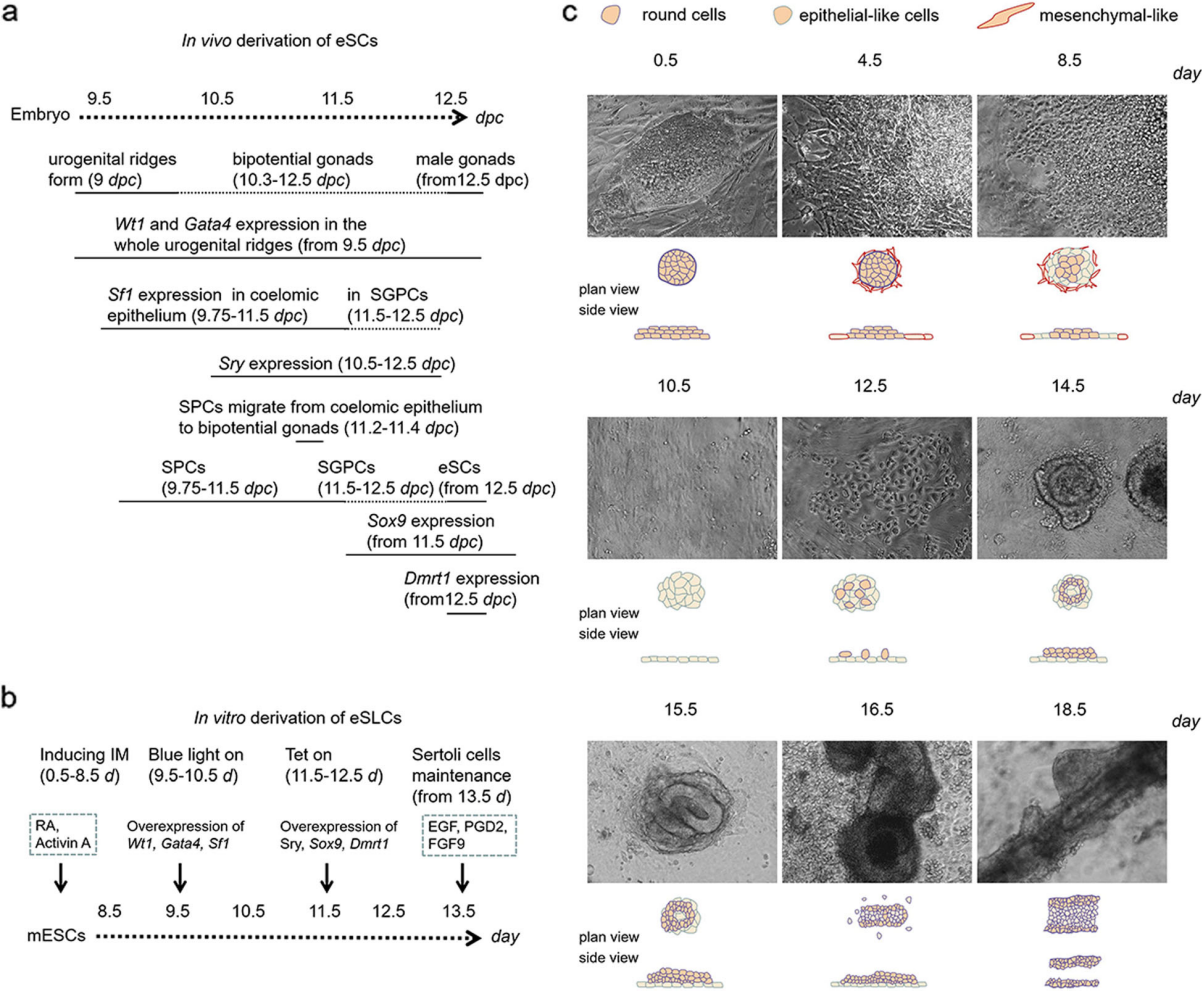
Establishment of a differentiation model. **a** A process map of in vivo derivation of embryonic Sertoli cells (eSCs). **b** Inducing strategies of in vitro derivation of embryonic Sertoli-like cells (eSLCs). **c** Optical micrographs of the cells generated and microstructures formed in the differentiation model in 18.5 days. The sketch of characteristic of generated cells was displayed below. Scale bar = 200 μm

In this inducing approach, mESCs were induced into IM through RA and Activin A in 0.5–8.5 days [26, 32, 46]. The overexpression of *Wt1*, *Gata4*, and *Sf1* was switched on via light-on system in 9.5–10.5 days. *Sox9* and *Dmrt1* were overexpressed in 11.5–12.5 days. Proteins of EGF, PGD2, and FGF9 were supplemented from 13.5 days to improve the maintenance of induced eSLCs (Fig. 1b). Results showed mESCs formed “pebble-like” colonies at 0.5 days (Fig. 1c). In 0.5–4.5 days, a great number of fibroblast-like and mesenchymal-like cells were generated along the edge of mESC colonies. In 4.5–8.5 days, the closely intercellular adhesion degenerated and these small round cells developed into epithelial-like cells. Around 10.5 days, most culture surface was covered by flat epithelium-like cells. In 10.5–12.5 days, some of the flat epithelial-like cells had EMT and turned into more solid form. In 12.5–14.5 days, these cells aggregated and formed into ring-like structures. In the following days, these ring-like structures continued their growth and eventually developed into tubular-like structures. Additionally, this developed process accorded with the presumptive in vivo cellular morphological changes from coelomic epithelial somatic cells to eSCs referring to the existing studies [6, 15, 16, 22, 23, 47].

**Fig. 2:**
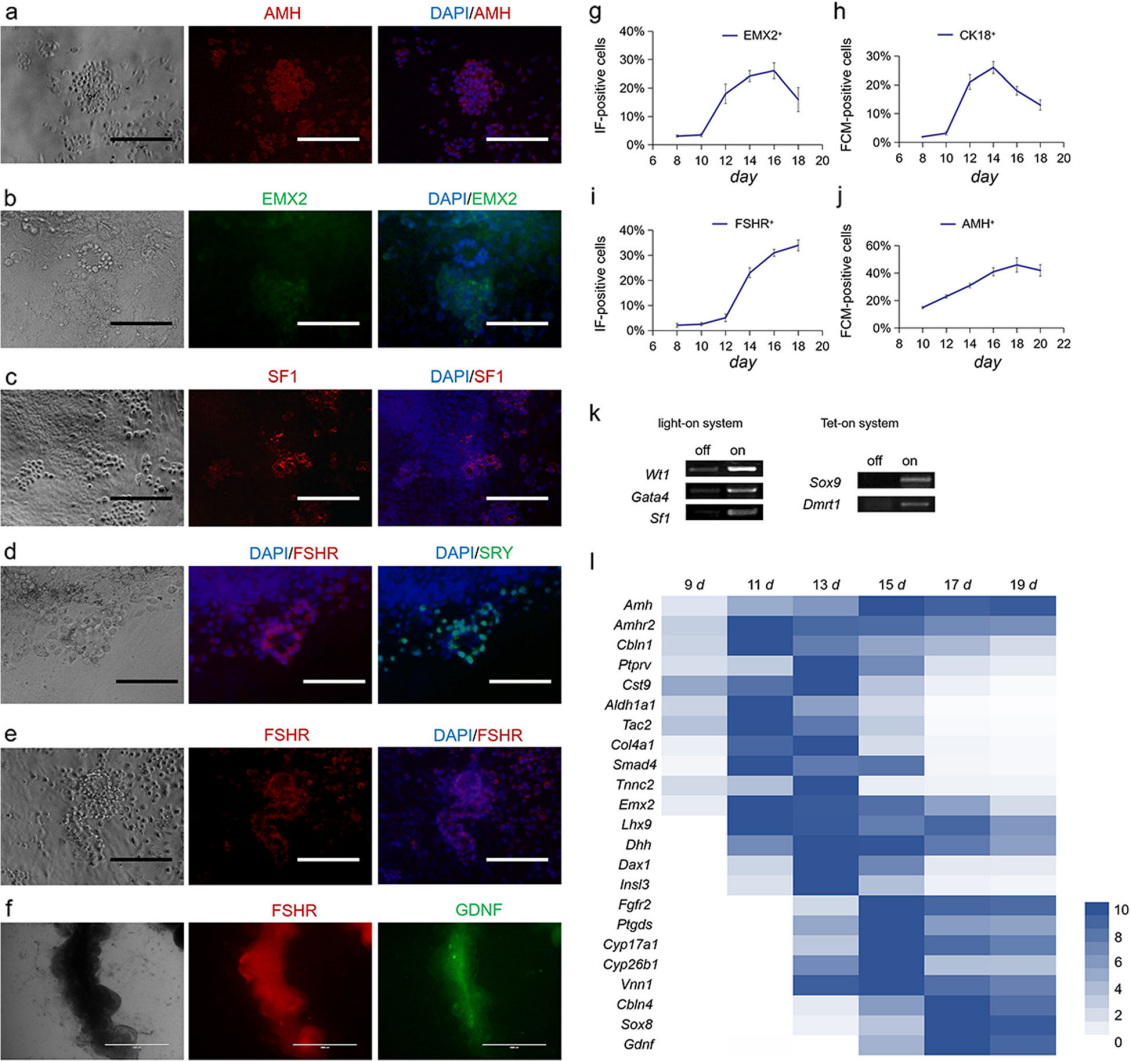
Marker identification and transcriptional determination of the cells in the differentiation model. Optical micrographs were displayed on the left. Immunofluorescence (IF) micrographs were on the right. **a** AMH^+^ cells were detected. **b** EMX2^+^ cells were detected. The cells of a ring-like structure were EMX2^−^. **c** SF1^+^ cells showed more solid than the epithelial-like cells around. **d***Fshr*^+^ cells showed ring-like microstructure. SRY^+^ cells were detected. **e** FSHR^+^ cells showed tubular-like microstructure. **f** FSHR^+^ and GDNF^+^ cells showed tubular-like microstructure. DAPI was a nuclear dye showing blue. Scale bar = 200 μm. The cell portion of **g** EMX2^+^ and **i** FSHR^+^ cells in differentiation model were determined via IF. The results took a mean value of three parallel experiments (20 views per sample) and were expressed as mean ± SD. The cell portion of **h** CK18^+^ and **j** AMH^+^ cells in differentiation model was determined via flow cytometry (FCM). Results were expressed as mean ± SD (*n* = 3 independent experiments). **k** The five target factors were independently expressed according to the procedures in the differentiation model. The transcriptional expression of target factors was detected through nucleotide band amplified by qPCR in 30 cycles. **l** Heat map indicated the transcriptional expression of stage-specific markers in the differentiation model by qPCR. Results took the mean value of qPCR (*n* = 3 independent experiments) and showed changes in gene expression relative to the highest expression in 9–19 days. The multiple ranged from 0 to 10

Furthermore, in order to define the differentiation process, some stage-specific markers were determined through IF, FCM, and qPCR. At 16 days, via IF, some suspected eSLCs possess a high expression of AMH (Fig. 2a). A ring-like structure colonies showed EMX2^−^ while the epithelial-like cells around showed EMX2^+^ (Fig. 2b). The cells constructing the ring-like structure were very likely to be eSLCs, and the epithelial-like cells around were speculated as coelomic epithelial somatic-like cells. The solid cells derived from flat epithelial-like cells expressed high in SF1 (Fig. 2c). Therefore, these were speculated as SPLCs or SGPLCs. In mice embryos, *Sry* was expressed in SGPCs in 10.5–12.5 days *pc*. At 14 days, some SRY^+^ cells tended to form a ring-like structure (Fig. 2d). Follicle-stimulating hormone receptor (FSHR) was a key gonadal specific marker which expressed either in eSCs or follicular cells. The FSHR^+^ cells forming a tubular-like structure were suspected as eSLCs (Fig. 2e). GDNF was expressed in eSCs. FSHR and GDNF had different expression level spatially in a tubular-like structure (Fig. 2f). These tubular-like structures potentially were formed of eSLCs. Based on these stage-specific marker, the cells were determined according to the characteristic of coelomic epithelial somatic cells, SPCs, SGPCs, and eSCs.

IF and FCM were performed every 2 days during determining the expression of EMX2, cytokeratin 18 (CK18), FSHR, and AMH. Results indicated the EMX2^+^ cells increased in 10–16 days (Fig. 2g). CK18^+^ cells raised in number in 10–14 days (Fig. 2h). In 12–18 days, the ratio of FSHR^+^ and AMH^+^ cells gradually increased (Fig. 2i, j). EMX2 was expressed in partial coelomic epithelial cells; CK18 was highly expressed in SGPCs; FSHR and AMH had high expression in SGPCs and eSCs. These markers implied the coelomic epithelial somatic-like cells, SGPLCs, and eSLCs could have been induced in 10–18 days.

Based on the transcriptional expression of specific marker genes related to coelomic epithelium development, gonadogenesis, and male determination, a heatmap of transcriptional changes in this differentiation model in 9–19 days was mapped (Fig. 2l). These results indicated that the marker genes related to coelomic epithelial somatic cells and their development including cerebellin 1 precursor protein (*Cbln1*), protein tyrosine phosphatase, receptor type, V (*Ptprv*), cystatin 9 (*Cst9*), aldehyde dehydrogenase family 1, subfamily A1 (*Aldh1a1*), tachykinin 2 (*Tac2*), collagen, type IV, alpha 1 (*Col4a1*), SMAD family member 4 (*Smad4*), troponin C2, fast (*Tnnc2*), *Emx2*, *Lhx9*, and insulin-like 3 (*Insl3*) were expressed high in 11–15 days. The marker genes related to eSCs including *Fgfr2*, *Ptgds*, Cytochrome P450, family 17, subfamily a, polypeptide 1 (*Cyp17a1*), Cytochrome P450, family 26, subfamily b, polypeptide 1 (*Cyp26b1*), vanin 1 (*Vnn1*), cerebellin 4 precursor protein (*Cbln4*), *Sox8*, and *Gdnf* had high expression after 15 days [14–16].

Via cellular morphology, specific markers and genetic transcription, and the development process were determined in this differentiation model. Then, it is crucial to determine whether the induced eSLCs had similar characteristic and function with eSCs. Some research had isolated mouse Sertoli-like cells by FSHR surface marker [25]. However, no FCM antibody of mouse FSHR on the market or other solid specific surface marker was found for isolating eSCs. Thus, the exact transcriptional and biomarker determination of the induced eSLCs was inaccessible. However, these eSLCs were determined in some other ways.

In our former paper “Differentiation Roadmap of Embryonic Sertoli Cells Derived from Mouse Embryonic Stem Cells” on “stem cell research & therapy”, the eSLCs were determined by markers including AMH and FASL. Observed under IF, some AMH^+^ epithelial-like cells were induced by transduction of the key factors (Additional file 8: Fig. S2c). In transduced group (mES + Trans), there were many FASL^+^ cells around the cell colony while the control group (mES + MEF) had negative result (Additional file 8: Fig. S2d). And some FASL^+^ cells formed a tubular-like structure and a ring-like structure (Additional file 8: Fig. S2e, f). Thus, these cells expressed the two specific biomarkers of eSCs and had similar morphological characteristic. Then, these FASL^+^ cells were sorted by FCM, stained by PKH26 dye, as well as some mature Sertoli cells, and injected into mice seminiferous tubules (ST). The images showed these FASL^+^ cells integrated into the ST without excessive aggregation (Additional file 9: Fig. S3A, B). And the immunohistochemistry result of the slices of ST indicated the spermatogonia stem cells (SSCs) (DDX4^+^) and sperm (PGP9.5^+^) suffered no obvious negative influence caused by these injected FASL^+^ cells. Results showed these FASL^+^ cells had similar physiological characteristics with Sertoli cells. To further determine these induced cells, the FASL^+^ cells and SSCs were co-cultured. These FASL^+^ cells expressed 91.1% of AMH^+^ and 53.3% of SOX9^+^ (Additional file 10: Fig. S4A, B). Thus, these FASL^+^ cells potentially included eSLCs and some other cells. The SSCs were isolated by Percoll density gradient centrifugation. In a-week coculture, some sperm-like cells were observed which could be derived from the SSCs (Additional file 10: Fig. S4F). These sperm-like cells showed PGP9.5^+^ and had long and narrow cell nucleus. Thus, the induced eSLCs could be functional in facilitating the maturation of SSCs. However, these sperm-like cells have not been determined by other important specific markers. Thus, there were still some evidences required to determine whether these eSLCs could support the SSCs.

Via FCM, IF, qPCR, and morphological identification, the differentiation process from mESCs to eSLCs was determined and these eSLCs had similar characteristic and expression of specific biomarkers with eSCs. Thus, the approach of inducing eSLCs could provide a platform for molecular mechanism research.

### *Wt1*, *Gata4*, and *Sf1* facilitated the generation of SPCs from IM

**Fig. 3:**
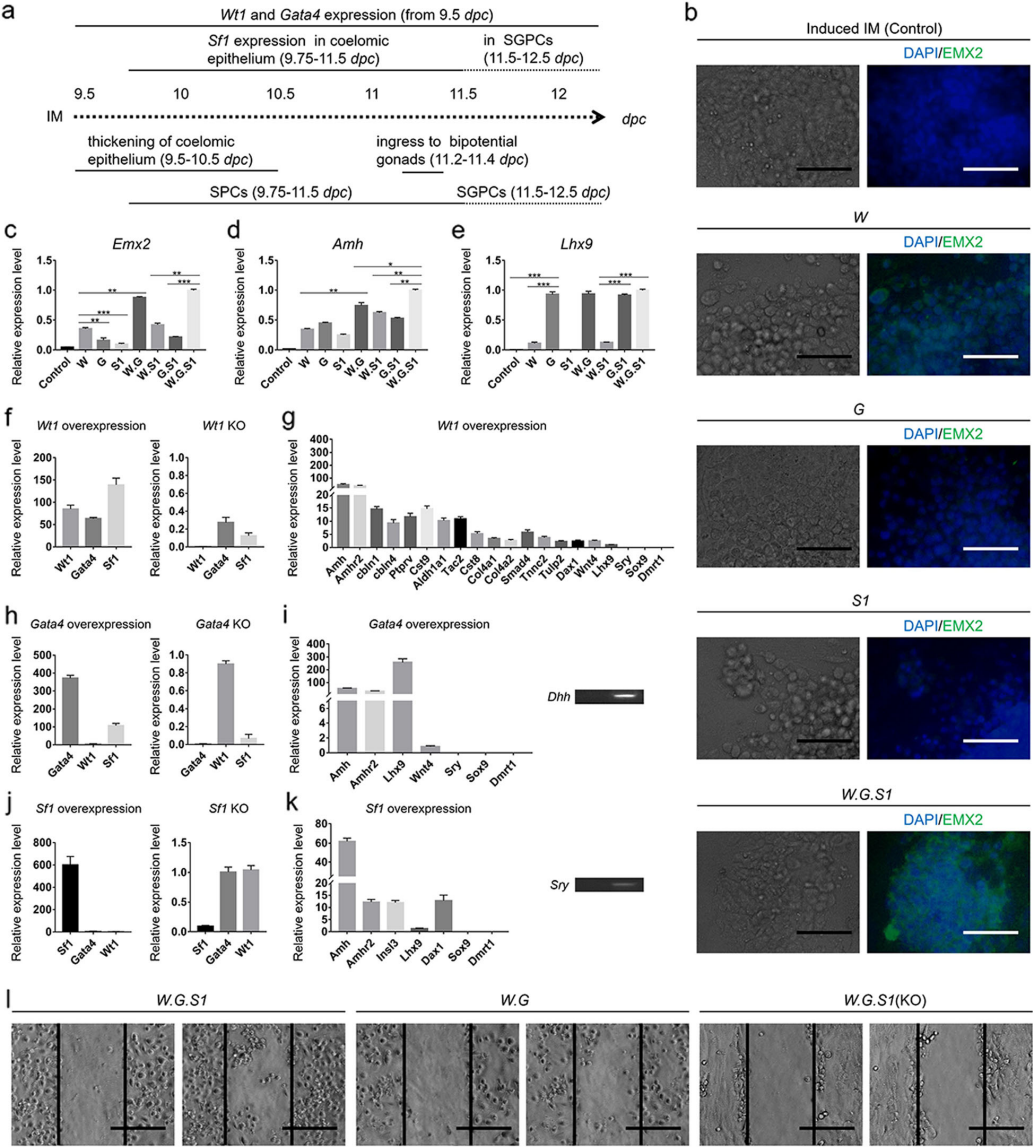
Determination of molecular mechanism in coelomic epithelium development stage. **a** A schematic diagram of gonadogenesis in 9.5–12 *dpc*. **b** Optical and IF micrographs were taken in different test groups at 12 days. DAPI staining showed blue in cell nucleus. EMX2^+^ cells showed green. Group (Induced IM) was the cells of differentiation model without genetic modification. Group (*W*) was the cells of differentiation model with overexpression of *Wt1*. Group (*W.G.S1*) was the cells of differentiation model with overexpression of *Wt1*, *Gata4*, and *Sf1*. The rest of the groups in this paper were done in the same manner. Scale bar = 200 μm. The transcriptional levels of **c***Emx2*, **d***Amh*, and **e***Lhx9* were determined in different groups. Results were expressed relative to the highest mean value as mean ± SD (*n* = 3 independent experiments). Asterisks indicate statistical significance of differences in the mean gene expression calculated by one-way ANOVA method. (*P value < 0.05, **P value < 0.01, ***P value < 0.001). The transcriptional levels in different group when **f***Wt1* was overexpressed or knock out (KO), **h***Gata4* was overexpressed or KO, and **j***Sf1* was overexpressed or KO were determined by qPCR at 12 days. Results were expressed related to the mean value of control group (group (Induced IM)) as mean ± SD (*n* = 3 independent experiments). The transcriptional levels when **g***Wt1* was overexpressed, **i***Gata4* was overexpressed, and **k***Sf1* was overexpressed were determined by qPCR at 12 days. Results were expressed related to the mean value of control group (group (Induced IM)) as mean ± SD (*n* = 3 independent experiments). In **i** and **k**, nucleotide band of *Dhh* and *Sry* was detected. The band of the control group was on the left. The band of test group was on the right. **l** Cell wound scratch assay. Optical micrographs showed the cells right after scratch on the left, and the cells at the second day on the right. Scale bar = 200 μm

In mice embryos, SPCs in coelomic epithelium were the main presumptive precursor of Sertoli cells [14, 16, 22]. The most accepted theory was that these SPCs were derived from coelomic epithelium at the side of bipotential gonad [23]. EMX2 expressed in coelomic epithelium was also located at the side of genital ridge formation (the precursor organ of bipotential gonad) [48]. Therefore, some of the SGPCs were speculated to be derived from these EMX2^+^ cells [6, 22, 48]. Based on existing studies, *Wt1*, *Gata4*, and *Sf1* were involved in this developmental phase (Fig. 3a). To reproduce it, three key factors, *Wt1*, *Gata4*, and *Sf1*, were overexpressed by different combinations through light-on transgene system in 9.5–10.5 days. The results of IF performed at 12 days indicated that the cells in the control group (induced IM), group (*S1*) (overexpression of *Sf1*), or group (*G*) (overexpression of *Gata4*) showed EMX2^*−*^ (Fig. 3b). Through overexpression of *Wt1* (group (*W*)), EMX2^+^ cells were observed. And then, under co-overexpression of *Wt1*, *Gata4*, and *Sf1*, the expression of EMX2 was tremendously improved. Via qPCR, the expression of *Emx2*, *Amh*, and *Lhx9* was determined in different groups at 12 days. Results were expressed relative to the highest value in all groups. The transcriptional expression of *Emx2* was mainly improved by the overexpression of *Wt1* (Fig. 3c). *Gata4* and *Sf1* facilitated the expression of *Emx2*. *Amh* was upregulated by *Wt1*, *Gata4*, or *Sf1* in different levels (Fig. 3d). *Lhx9* had a major upregulated expression by *Gata4* and a minor upregulated expression by *Wt1* (Fig. 3e). *Emx2* and *Lhx9* were the specific markers of the SPCs. Thus, the results indicated *Wt1* and *Gata4* play major roles in inducing the SPLCs, and overexpression of *Sf1* had a slight positive effect. However, these results could have been influenced by the interaction among *Wt1*, *Gata4*, and *Sf1*. Through genetic overexpression or KO, the transcriptional results indicated the overexpression of *Wt1* enhanced the expression of *Gata4* and *Sf1* (Fig. 3f). The overexpression of *Gata4* improved the expression of *Sf1*, but not *Wt1* (Fig. 3h). The overexpression of *Sf1* had no obvious influence on *Wt1* or *Gata4* (Fig. 3j). Inversely, *Wt1* KO caused decrease expression on *Gata4* and *Sf1* (Fig. 3f). KO of *Gata4* mainly inhibited the expression of *Sf1* (Fig. 3h). Furthermore, *Sf1* KO took little influence on *Wt1* or *Gata4* (Fig. 3j). Conclusively, in the cells of induced IM, *Gata4* and *Sf1* was upregulated by overexpression of *Wt1*, and *Sf1* was upregulated by overexpression of *Gata4*. These interactions influenced the expression of *Emx2*, *Amh*, or *Lhx9* in test groups and caused difficulties to distinguish the individual role of *Wt1*, *Gata4*, or *Sf1* in this developmental phase. To further identify their roles, some other relevant genes were transcriptionally determined. Overexpression of *Wt1* greatly improved the expression of *Amh* and anti-Mullerian hormone type 2 receptor (*Amhr2*), obviously enhanced the expression of *Cbln1*, *Cbln4*, *Ptprv*, *Cst9*, and *Tac2*, and slightly influenced the expression of cystatin 8 (*Cst8*), *Col4a1*, collagen, type IV, alpha 2 (*Col4a2*), *Smad4*, *Tnnc2*, tubby-like protein 2 (*Tulp2*), nuclear receptor subfamily 0, group B, member 1 (*Dax1*), and wingless-type MMTV integration site family, member 4 (*Wnt4*) (Fig. 3g) [49]. When *Gata4* was overexpressed, the transcriptional expression of *Amh*, *Amhr2*, and *Lhx9* greatly increased. Desert hedgehog (*Dhh*) was activated (Fig. 3i). In *Sf1* overexpression group, the expression of *Amh* had major upregulation, and *Amhr2*, *Insl3*, and *Dax1* had minor upregulation (Fig. 3k). The transcription of *Sry* was barely detected in qPCR with 30 amplification cycle and *Sox9* was unobserved. According to these results, *Wt1* and *Gata4* activated many important genes related to the development of coelomic epithelial somatic cells and SPCs, and *Sf1* did not show an obvious role. However, through wound healing assay, it was observed that more cells in group (*W.G.S1*) migrated to the scratched surface than those in group (*W.G*) (Fig. 3l). It indicated the overexpression of *Sf1* improved the cell migration. In group (*W.G.S1*(KO)), most of the cells showed epithelial-like morphology and migrated much slower than the other two groups. The results indicated KO of *Sf1* potentially impeded the generation of SPLCs. Then, *Sf1* was supposed to play important roles in EMT, migration, and differentiation of coelomic epithelial somatic-like cells to SPCs.

In this study, *Wt1*, *Gata4*, and *Sf1* were determined on their roles in the derivation of SPCs with basic molecular mechanism. These results provided evidences to map the molecular pathways in the phase of the development of coelomic epithelium.

### Manual onset of male determination is necessary for inducing eSLCs in this differentiation model

**Fig. 4:**
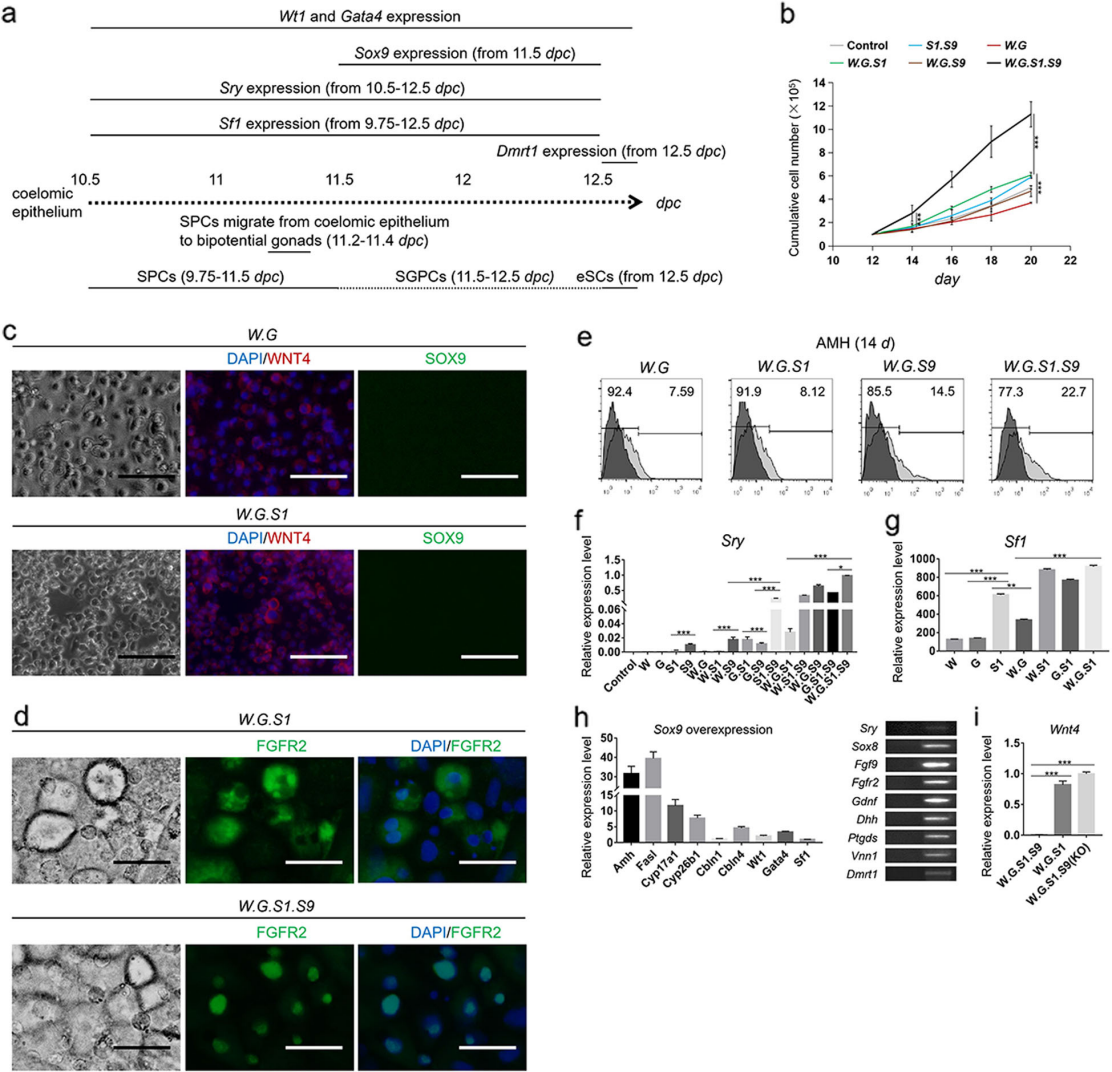
Determination of molecular mechanism in male determination and generation of eSCs. **a** A schematic diagram of derivation of eSCs in 10.5–12.5 *dpc*. **b** Growth curves of different groups in 12–20 days. Results were expressed as mean ± SD (*n* = 3 independent experiments). Asterisks indicate statistical significance of difference in the mean cell number calculated by one-way ANOVA method. (*P value < 0.05, **P value < 0.01, ***P value < 0.001). **c** Optical and IF micrographs were taken in groups (*W.G*) and (*W.G.S1*) at 14 days. DAPI staining showed blue in cell nucleus. WNT4^+^ cells showed red. SOX9^+^ cells showed green. Scale bar = 200 μm. **d** Optical and IF micrographs were taken in groups (*W.G.S1*) and (*W.G.S1.S9*) at 14 days. DAPI staining showed blue in cell nucleus. FGFR2^+^ cells showed green. Scale bar = 20 μm. **e** AMH^+^ cells were determined via FCM in different groups at 14 days. The control peak took the result of isotype antibody showing dark color. The positive result refers to the control result. **f** The transcriptional levels of *Sry* were determined in different groups. Results were expressed relative to the highest mean value as mean ± SD (*n* = 3 independent experiments). Asterisks indicate statistical significance of differences in the mean gene expression calculated by one-way ANOVA method. (*P value < 0.05, **P value < 0.01, ***P value < 0.001). **g** The transcriptional levels of *Sf1* were determined in different groups. Results were expressed relative to the result of control group (group (Induced IM)) at 14 days as mean ± SD (*n* = 3 independent experiments). Asterisks indicate statistical significance of differences in the mean gene expression calculated by one-way ANOVA method. (*P value < 0.05, **P value < 0.01, ***P value < 0.001). **h** The transcriptional levels when *Sox9* was overexpressed were determined by qPCR at 14 days. Results were expressed related to the mean value of control group (group (Induced IM)) as mean ± SD (*n* = 3 independent experiments). Nucleotide band of relevant genes was detected. The band of control group was on the left. The band of test group was on the right. **i** The transcriptional levels of *Wnt4* were determined in different groups. Results were expressed relative to the highest mean value as mean ± SD (*n* = 3 independent experiments). Asterisks indicate statistical significance of differences in the mean gene expression calculated by one-way ANOVA method. (*P value < 0.05, **P value < 0.01, ***P value < 0.001)

In mice embryos, some coelomic epithelial somatic cells developed into SPCs, underwent an EMT, ingressed into bipotential gonad, became SGPCs, and finally differentiated into eSCs under the influence of factors including *Wt1*, *Gata4*, *Sf1*, *Sry*, and *Sox9*. (Fig. 4a). To investigate the molecular mechanism, the key factors were tested in different combination and manipulated according to the schedule of the established differentiation model.

Generally, eSCs have a high proliferation ability [50, 51]. The growth curve of different groups potentially indicated the generation of eSCs. In group (*W.G.S1.S9*), the cumulative cell number increased much faster than the other groups (Fig. 4b). Thus, it was speculated that eSLCs have been successfully induced in group (*W.G.S1.S9*). To verify this opinion, the cells were determined by WNT4 and SOX9 markers via IF in different test groups. Results showed SOX9 was not detected under the overexpression of *Wt1*, *Gata4*, and *Sf1* (Fig. 4c). On the contrary, the expression of female determining factor WNT4 expressed higher in group (*W.G.S1*) than in group (*W.G*). These results indicated that the overexpression of *Wt1*, *Gata4*, and *Sf1* was not sufficient to initiate male determination in this differentiation model. On the other hand, overexpression of *Sf1* even promoted the expression of female determining factors. To demonstrate it, FGFR2 was detected via IF. FGFR2 was expressed in nucleoplasm of eSCs, or in cytoplasm of follicular cells [37]. Thus, based on the domain of FGFR2 protein, it indicated follicular-like cells were induced in group (*W.G.S1*), and eSLCs were induced in group (*W.G.S1.S9*) (Fig. 4d). According to the results at 14 days, the ratio of AMH^+^ cells was 22.7%, 14.5%, 8.12%, and 7.59% respectively in group (*W.G.S1.S9*), (*W.G.S9*), (*W.G.S1*), and (*W.G*) (Fig. 4e). AMH was expressed low in SPCs, SGPLCs, follicular cells, and much higher in eSCs [6, 23, 52]. These results implied the overexpression of *Sox9* altered the fate of SGPCs into eSCs which proliferated rapidly and expressed high in AMH.

*Sry* gene plays a key role in the onset of male determination and an important specific marker of eSCs [53, 54]. In mice embryos, *Sry* was expressed in 10.5–12.5 *dpc*, following the expression of *Wt1*, *Gata4*, and *Sf1* [55]. However, in this differentiation model, *Sry* was not activated under the overexpression of these three factors (Table 1). To demonstrate it, the transcriptional levels of *Sry* were determined in different groups. Co-overexpression of *Wt1*, *Gata4*, and *Sox9* (group (*W.G.S9*)) and *Wt1*, *Gata4*, *Sf1*, and *Sox9* (*W.G.S1.S9*) efficiently activated the expression of *Sry* (Fig. 4f). However, *Sf1* can also be upregulated by the overexpression of *Wt1* and *Gata4* (Fig. 4g). Thus, these results implied high expression of *Sry* potentially depended on the co-overexpression of *Wt1*, *Gata4*, *Sf1*, and *Sox9*. In factor analysis results via IF, results showed the SRY^+^ cells were observed in group (*S1.S9*), (*S1.S9.D*), (*G.S1.S9*), (*W.S1.S9*), (*W.G.S9*), (*W.G.S1.S9*), (*W.G.S9.D*), (*W.S1.S9.D*), (*G.S1.S9.D*), and (*W.G.S1.S9.D*) (Table 1). These results indicated the minimum factors to activate *Sry* were *Sf1* and *Sox9*. In addition, the overexpression of *Dmrt1* did not show obvious influence on the expression of *Sry*. Via qPCR, *Wnt4* was inhibited in group (*W.G.S1.S9*) (Fig. 4i). However, *Wnt4* was highly expressed when *Sox9* was not manually overexpressed in group (*W.G.S1*) or knocked out in group (*W.G.S1.S9*(KO)). The results indicated that overexpression of *Sox9* was essential to inhibit the female determination in this differentiation model. Transcriptional results of overexpression of *Sox9* were determined at 14 days and expressed relative to the control group (Induced IM without genetic modification). *Amh* and *Fasl* had major increase (Fig. 4h). *Cyp17a1*, *Cyp26b1*, *Cbln4*, and *Gata4* had minor increase. In addition, some male determining factors including *Sox8*, *Fgf9*, *Fgfr2*, *Gdnf*, *Dhh*, *Ptgds*, *Vnn1*, and *Dmrt1* were detected [5, 17, 47].

**Table 1:** Factor analysis of *Wt1*, *Gata4*, *Sf1*, *Sox9*, and *Dmrt1* in the differentiation model

	E	S	F	W		E	S	F	W
5 Factors					2 Factors				
*W.G.S1.S9.D*	+	+	+	–	*W.G*	+	–	+	+
4 Factors					*W.S1*	+	–	+	+
*G.S1.S9.D*	+	+	+	–	*W.S9*	+	–	+	–
*W.S1.S9.D*	+	+	+	–	*W.D*	+	–	–	+
*W.G.S9.D*	+	+	+	–	*G.S1*	–	–	–	+
*W.G.S1.D*	+	–	+	+	*G.S9*	–	–	–	–
*W.G.S1.S9*	+	+	+	–	*G.D*	–	–	–	+
3 Factors					*S1.S9*	–	+	+	–
*W.G.S1*	+	–	+	+	*S1.D*	–	–	+	+
*W.G.S9*	+	+	+	–	*S9.D*	–	–	–	–
*W.G.D*	+	–	+	+	1 Factor				
*W.S1.S9*	+	+	+	–	*W*	+	–	–	–
*W.S1.D*	+	–	+	+	*G*	–	–	–	–
*W.S9.D*	+	–	+	–	*S1*	–	–	–	–
*G.S1.S9*	–	+	–	–	*S9*	–	–	–	–
*G.S1.D*	–	–	–	+	*D*	–	–	–	–
*G.S9.D*	–	–	–	–					
*S1.S9.D*	–	+	–	–					

*E* EMX2, *S* SRY, *F* FSHR, *W* WNT4

In this differentiation model, it was found that the male determination was not sufficiently activated under the co-overexpression of *Wt1*, *Gata4*, and *Sf1* and initiated by additional overexpression of *Sox9*. However, the onset of *Sry* was following the expression of *Wt1*, *Gata4*, and *Sf1*, followed by *Sox9* in vivo development. Thus, these results further raised the question of reproducing the molecular pathways of male determination in differentiation model.

### *Dmrt1* improved the cell proliferation and the microstructure formation of eSCs

**Fig. 5:**
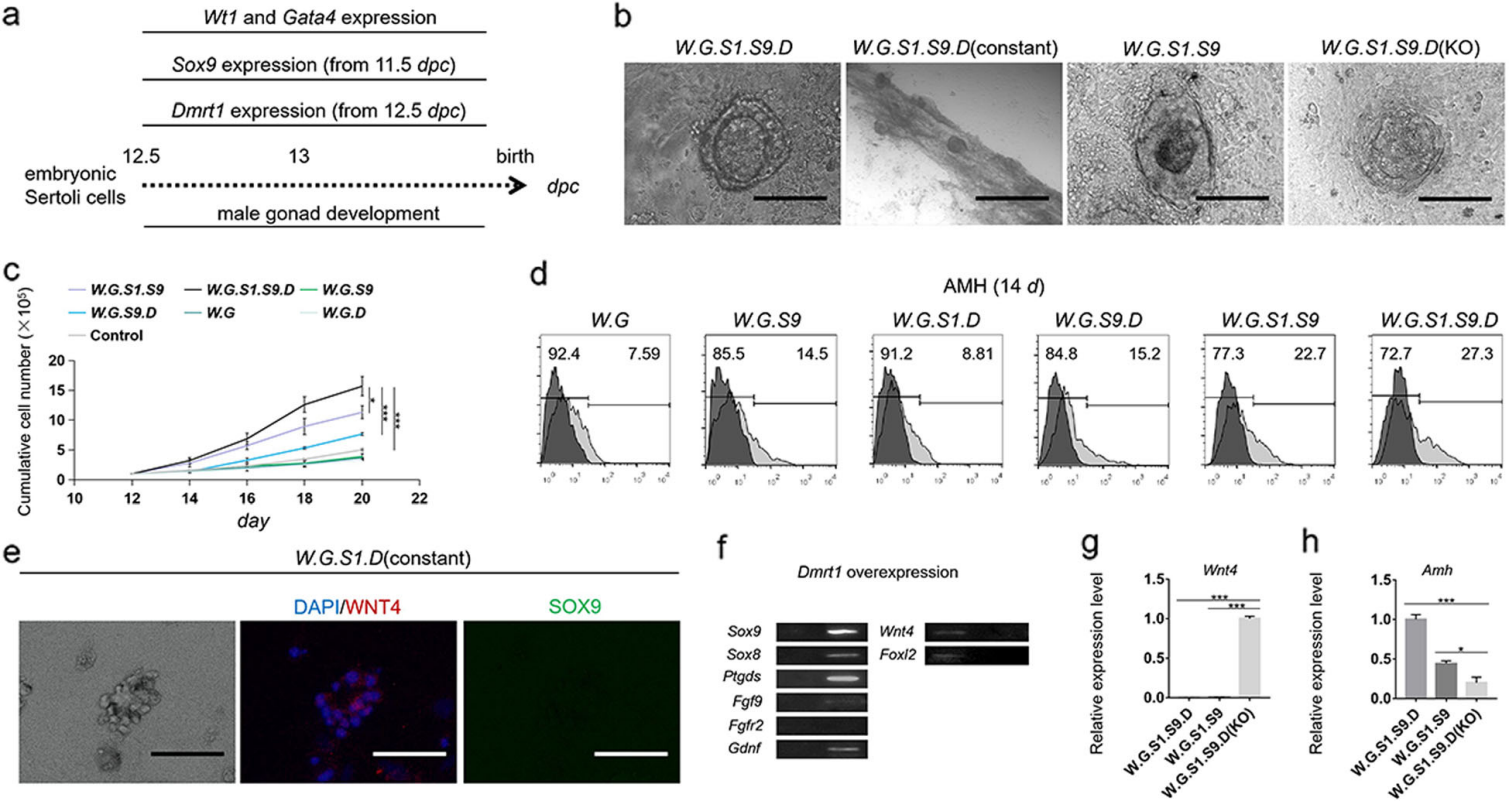
Determination of molecular mechanism in maintenance of eSCs. **a** A schematic diagram of maintenance of eSCs from 12.5 *dpc*. **b** Optical micrographs showed ring-like or tubular-like microstructures in different group. **c** Growth curves of different groups in 12–20 days. Results were expressed as mean ± SD (*n* = 3 independent experiments). Asterisks indicate statistical significance of difference in the mean cell number calculated by one-way ANOVA method. (*P value < 0.05, **P value < 0.01, ***P value < 0.001). **d** AMH^+^ cells were determined via FCM in different groups at 14 days. The control peak took the result of isotype antibody showing a dark color. The positive result refers to the control result. **e** Optical and IF micrographs were taken in group (*W.G.S1.D*(constant)) in 20 days. DAPI staining showed blue in cell nucleus. WNT4^+^ cells showed red. SOX9^+^ cells showed green. Scale bar = 200 μm. **f** Nucleotide band of relevant genes was detected at 14 days when Dmrt1 was overexpressed. The band of control group was on the left. The band of test group was on the right. The transcriptional levels of **g***Wnt4* and **h***Amh* were determined in different groups. Results were expressed relative to the highest mean value as mean ± SD (*n* = 3 independent experiments). Asterisks indicate statistical significance of differences in the mean gene expression calculated by one-way ANOVA method. (*P value < 0.05, **P value < 0.01, ***P value < 0.001)

In mice gonadal development, *Dmrt1* was expressed since 12.5 *dpc*, later than all the other four key factors (Fig. 5a). Based on previous study, *Dmrt1* mainly played its role in the maintenance of male gonadal development [56, 57]. The deficiency of *Dmrt1* caused sexual reversal [19, 58]. In this differentiation model, some similar phenomena were observed.

To determine the role of *Dmrt1*, the eSLCs formed ring-like structures under co-overexpression of *Wt1*, *Gata4*, *Sf1*, *Sox9*, and *Dmrt1*, but failed to form a tubular-like structure (Fig. 5b). In group (*W.G.S1.S9.D*(constant)), *Wt1*, *Gata4*, *Sf1*, and *Sox9* were upregulated according to the differentiation model, and *Dmrt1* was constantly overexpressed. Optical micrographs showed ring-like and tubular-like structures were successfully formed in 20 days. In the absence of the overexpression of *Dmrt1*, ring-like structures had degeneration and did not develop into tubular-like structures in group (*W.G.S1.S9*). When *Dmrt1* was knocked out, the presumptive eSLCs could hardly form a ring-like structure in group (*W.G.S1.S9.D*(KO)). These results indicated the expression of *Dmrt1* influenced the microstructure formation of eSLCs. On the contrary, there was ring-like structure observed in group (*W.G.S1.D*(constant)) and these cells showed WNT4^+^ and SOX9^−^ (Fig. 5e). Supposedly, these WNT4^+^/SOX9^−^ cells were not eSLCs; however, the overexpression of *Dmrt1* had given these somatic cells a similar behavior as eSCs.

Via FCM, the ratio of AMH^+^ cells was 27.3% in group (*W.G.S1.S9.D*), 22.7% in group (*W.G.S1.S9*), 15.2% in group (*W.G.S9.D*), 14.5% in group (*W.G.S9*), 8.81% in group (*W.G*), and 7.59% in group (*W.G.S1.S9.D*) (Fig. 5d). In comparison to groups (*W.G.S1.S9.D*) and (*W.G.S1.S9*), it indicated the overexpression of *Dmrt1* could improve the generation of AMH^+^ cells. The growth curve of these groups indicated the cell number of group (*W.G.S1.S9.D*) was close to that of group (*W.G.S1.S9*) in 12–16 days, and then much higher than that of group (*W.G.S1.S9*) in 18–20 days (Fig. 5c). These results indicated the overexpression of *Dmrt1* improved the cell proliferation. In combination of the results of FCM and growth curve, *Dmrt1* was proven to play important roles in improving the cell proliferation and the generation of AMH^+^ cells. The ratio of AMH^+^ cells in group (*W.G.S1.S9.D*) (27.3%) was much higher than that in group (*W.G.S1.D*) (8.81%), and the ratio of AMH^+^ cells in group (*W.G.S1.S9*) (22.7%) was much higher than that in group (*W.G.S1*) (8.12%) (Figs. 4e and 5d). Based on the former inference, the overexpression of *Sox9* was essential for the SGPLCs to develop into eSLCs. Thus, these results implied *Dmrt1* potentially improved the ratio of AMH^+^ cells and cell proliferation by facilitating the generation of eSLCs.

To determine the molecular function of *Dmrt1*, it was overexpressed, activated the expression of *Sox9*, *Sox8*, *Ptgds*, *Fgf9*, and *Gdnf*, and inhibited *Wnt4* and forkhead box L2 (*Foxl2*) (Fig. 5f). Consequently, *Dmrt1* could activate factors related to male determination and development. In groups (*W.G.S1.S9.D*) and (*W.G.S1.S9*), *Wnt4* was inhibited (Fig. 5g). However, the expression of *Wnt4* was upregulated when *Dmrt1* was knocked out (*W.G.S1.S9.D*(KO)). These results indicated *Dmrt1* was essential for male maintenance even when *Sox9* has been activated. The expression of *Amh* decreased successively in groups (*W.G.S1.S9.D*), (*W.G.S1.S9*), and (*W.G.S1.S9.D*(KO)) (Fig. 5h). Thus, results indicated that overexpression of *Dmrt1* facilitated the transcriptional expression of *Amh* or generation of AMH^+^ cells, while KO of *Dmrt1* reduced them.

In conclusion, *Dmrt1* potentially played important roles in improving cell proliferation, facilitating the generation of AMH^+^ cells or expression of *Amh*, activation of the male determining factors, and promoting the ring-like microstructure formation of induced eSLCs.

## Discussion

### Evaluating the performance of established differentiation model

In embryonic development, urogenital ridges and coelomic epithelium were formed from IM at 9 *dpc* (Fig. 1a) [15]. Some of the coelomic epithelial somatic cells expressed SF1 since 9.75 days, underwent EMT, ingressed into bipotential gonads in 11.2–11.4 *dpc*, and became SGPCs [14]. In 11.5–12.5 *dpc*, these SGPCs went through mesenchymal-epithelial transition (MET) and developed into eSCs through male determination [16]. In this differentiation model, some similar developmental processes were reproduced. For example, a lot of epithelial-like cells have been generated at 10.5 days (Fig. 1c). Some of these cells had morphological changes similar to EMT in 10.5–12.5 days. Ring-like structures formed of eSLCs were constructed in 12.5–14.5 days. Then, tubular-like structures were formed in 14.5–18.5 days. Results of the expression of the specific markers indicated that the SPLCs and SGPLCs derived from coelomic epithelial somatic-like cells (EMX2^+^, CK18^+^) increased in 12–14 days (Fig. 2g, h). eSLCs (FSHR^+^, AMH^+^) grew in number in 14–18 days (Fig. 2i, j). So, the order of generated cells from SPCs to eSCs in embryos had been reproduced. However, the timeline of generation of SPCs (9.75–11.5 days), SGPCs (11.5–12.5 days), and eSCs (12.5 days) was delayed in this differentiation model. In this differentiation model, IM was induced by RA and Activin A in 0.5–8.5 days (Fig. 1b) [32]. *Wt1*, *Gata4*, and *Sf1* were upregulated in 9.5–10.5 days through a light-on system. *Sox9* and *Dmrt1* were overexpressed in 11.5–12.5 days through a Tet-on system. EGF, PGD2, and FGF9 were supplemented since 13.5 days. Correspondingly, in embryonic development, expression schedule of key factors was as follows: the *Wt1* and *Gata4* had an expression since 9.5 *dpc*. *Sf1* expressed in 9.75–12.5 *dpc*. *Sox9* maintained an expression from 11.5 *dpc*. *Dmrt1* began to express at 12.5 *dpc* (Fig. 1a). Thus, the genetic regulation in the differentiation model corresponded with the expression schedule during embryonic development. However, as mentioned before, the timeline of generation of SPCs, SGPCs, and eSCs was delayed. There could be several reasons: (1) The light-on and Tet-on system potentially required a certain response time to express the target genes in the cells of induced IM. To solve this problem, these target factors could be determined through qPCR and Western blot to confirm their actual working time point. Then, the manipulation of the factors could be regulated based on these results. (2) In embryonic development, *Sf1* expressed during 9.75–12.5 *dpc*. *Wt1*, *Gata4*, *Sox9*, and *Dmrt1* kept expression since they were activated. This differentiation model adopted the spatiotemporal genetic control, but did not perfectly reproduce the genetic changes in vivo. To deal with this, the expression of these key factors can be extended and completely accorded with the schedule in vivo. The overexpression of *Wt1*, *Gata4*, *Sox9*, and *Dmrt1* can be constantly activated using pLenti lentiviral vectors. *Sf1* can be upregulated during 9.75–12.5 *dpc* using the light-on transgene system. However, the constant overexpression of the key factors potentially causes improper genetic expression profiles and failure of cellular development. (3) The generation of SPCs, SGPCs, and eSCs from coelomic epithelium could be influenced by a specific micro-environment such as coelomic epithelium or bipotential gonad. In this differentiation model, the eSCs-inducing strategy was performed based on induced IM by RA signaling. Under the co-overexpression of *Wt1*, *Gata4*, and *Sf1*, it was hard to reproduce a micro-environment like coelomic epithelium. Hopefully, application of novel techniques, supplements, and cell scaffolds could potentially provide solutions.

In this differentiation model, the induced cells had presented some specific behaviors and characteristics. The induced SF1^+^ cells derived from the epithelial-like cells showed solid form were active in migration and aggregation (Fig. 2c). Correspondingly, SPCs showed high activity in migration when they ingressed from coelomic epithelium to bipotential gonad. Thus, these SF1^+^ cells were similar to the SPCs. In male gonad, eSCs formed scaffold of seminiferous tubules and supported the testicle. In this work, ring-like structures were formed and the induced eSLCs forming the ring-like structures showed FSHR^+^\EMX2^−^ as the same expression of specific biomarkers as eSCs in embryos (Figs. 1c and 2b). And then, these ring-like structures developed into tubular-like structures which reproduced the behavior of constructing seminiferous tubules (Figs. 1c and 2d, f). Thus, results indicated these induced eSLCs possessed a highly similar physiological behavior with the eSCs.

Conclusively, this differentiation model of inducing eSLCs from mESCs was successful in mimicking a similar timeline of generated cell types and cellular behaviors. Hopefully, this model could provide a new platform to investigate gonadogenesis, male determination, productive disorder, and toxicology.

### Mapping the molecular differentiation pathways from IM to eSCs

In this study, the major work was to clarify the molecular mechanism of deriving eSCs through a mESC differentiation model. Based on existing studies, *Wt1*, *Gata4*, *Sf1*, *Sry*, *Sox9*, and *Dmrt1* were speculated as the central factors in the formation of genital ridge and male gonad. In this work, the molecular functions and mechanism were determined.

*Wt1*, *Gata4*, and *Sf1* have complicated interactions. The overexpression of *Wt1* improved the expression of *Gata4*, *Sf1*, and other important factors expressed in coelomic epithelium including *Ptprv*, *Aldh1a1*, and *Tac2* (Fig. 3f, g). The upregulation of *Gata4* improved expression of *Sf1* and initiated the expression of two important factors respectively expressed in bipotential gonad and male gonad, *Lhx9* and *Dhh* (Fig. 3h, i). Overexpression of *Sf1* activated *Insl3*, *Dax1*, and *Sry*, but did not affect other important factors related to the development of coelomic epithelial development including *Emx2*, *Cbln1*, *Cbln4*, or *Cst9* (Fig. 3j, k). Via qPCR, the expression of *Emx2* and *Amh* was greatly improved with overexpression of *Wt1* or co-overexpression of *Wt1* and *Gata4*. Thus, it was speculated that the expression of *Wt1* activated most of the genes related to the development of coelomic epithelium and generation of SPCs. *Gata4* played its role in activating some important factors for generation of SPCs and male determination.

*Sf1* potentially carried forward the EMT of coelomic epithelial somatic cells, improved cell migration of SPCs and SGPCs, and played key role in sex determination. Via IF, results showed the SF1^+^ cells had morphological changes differ from coelomic epithelial somatic-like cells and were active in migration (Fig. 2c). Thus, *Sf1* was potentially functional in improving the EMT and migration. Based on the existing studies, *Sf1* had many upstream factors including *Wt1*, *Gata4*, *Lhx9*, *Emx2*, *Cbx2*, Insulin/Insulin-like growth factor (IGF) signaling, *Six*1/4, and FOG2. In the downstream, *Sf1* simultaneously facilitated the expression of male determining factors and female determining factor (Figs. 3k and 4c). Results showed overexpression of *Sf1* was insufficient to activate the expression of *Sry* by itself (Fig. 4g). Some studies indicated that some of the upstream factors of *Sf1* also played important roles in sex determination [59, 60]. Therefore, it is speculated that *Sf1* is a core intermediate factor to carry forward the earlier development stage to sex determination stage.

In the stage of SGPCs developing into eSCs, the key male determining factors, *Sry* and *Sox9*, were not sufficiently upregulated through co-overexpression of *Wt1*, *Gata4*, and *Sf1* (Fig. 4c, d, g). On the contrary, female determining factor, *Wnt4*, was expressed highly [38]. To activate *Sry*, overexpression of *Sox9* was essential in this differentiation model (Fig. 4d, g). However, *Sry* was expressed ahead of *Sox9* in embryonic development [61, 62]. These results raised the questions of the onset of *Sry* and molecular mechanism of male determination. In vivo, *Sry* (10.5 *dpc*) initiated after the onset of *Sf1* (9.75 *dpc*) and ceased at a same time point of *Sf1* (12.5 *dpc*). Thus, it was highly suspected that the expression of *Sry* required a high expression level of *Sf1* [63, 64]. Via IF, SRY was detected in presumptive eSLCs (Fig. 2d). Hypothetically, the expression of SRY was potentially limited to some defined cell types such as SGPCs or eSCs. Thus, the reason that *Sry* had not been activated under the co-overexpression of *Wt1*, *Gata4*, and *Sf1* may due to the insufficient period of genetic upregulation. In 9.5–10.5 days, the upregulation of the three target factors was initiated through the light-on system. There could be a preparation period for the target genes to transcribe, and then induce the SPLCs, SGPLCs, and eSLCs. Thus, the expression level of *Sf1* may have largely declined when these SGPLCs were induced and ready for sex determination. There were some chances that the onset of *Sry* requires a high expression level of *Sf1* in these SGPLCs. Thus, the expression of *Sry* was not successfully activated with co-overexpression of *Wt1*, *Gata4*, and *Sf1* in this differentiation model. To demonstrate this opinion, the future work would determine the transcriptional expression level and protein expression of SF1 in 9–15 days to reveal the connection between *Sf1* expression and onset of *Sry* in this established differentiation model.

Although *Wt1*, *Gata4*, and *Sf1* failed to initiate male determination in this approach, *Sry* was activated, and eSLCs were successfully induced via overexpression of *Sox9* (Figs. 1e and 4d, e). These cells possess high expression of FSHR and AMH, expressed FGFR2 in nucleoplasm, formed ring-like and tubular-like microstructure (Figs. 1c and 2d, e, f, i, j). Results indicated the overexpression of *Sox9* was essential for the SGPLCs to develop into eSLCs instead of follicular-like cells. Through transcriptional determination, *Sox9* improved the expression of testicular development related factors including *Amh*, *Fasl*, *Cyp17a1*, *Cyp26b1*, *Cbln4*, *Wt1*, and *Gata4* and inhibited female determining factors *Wnt4* (Fig. 4h, i). Thus, *Sox9* played an important role in initiating the male determination and inhibiting the female determination in this differentiation model. Some studies showed *Sox9* is sufficient for functional testis development producing fertile male mice in the absence of S*ry* [65]. In this differentiation model, the SRY^+^ cells were observed; however, not all the eSLCs showed SRY^+^ (Fig. 2d). Thus, some SGPCs potentially developed into eSCs directly under the influence of *Sox9* without the onset of *Sry*.

*Dmrt1* also inhibited the female determining factors including *Wnt*4 and *Foxl2* (Fig. 5f) [19, 66]. The absent overexpression of *Dmrt1* did not prevent the generation of eSLCs (Figs. 4d and 5d). Thus, *Dmrt1* mainly played roles in maintaining eSLCs instead of inducing them [63]. Results indicated that *Dmrt1* affected the organ formation of the induced eSLCs (Fig. 5b). Without overexpression of *Dmrt1* or supplement of PGD2, FGF9, FGFR2, or GDNF, the ring-like structures degenerated and failed to form tubular-like structure in group (*W.G.S1.S9*). PGD2, FGF9, FGFR2, or GDNF were expressed in male gonad and facilitated the development of eSCs [39, 67–70]. Without these factors, the overexpression of *Dmrt1* helped the eSLCs to form a ring-like structure in group (*W.G.S1.S9.D*). And in a long-term overexpression of *Dmrt1*, tubular-like structures were successfully formed in group (*W.G.S1.S9.D*(constant)). Via transcriptional determination, overexpression of *Dmrt1* transcriptionally activated the expression of *Ptgds*, *Fgf9*, and *Gdnf* (Fig. 5f). Thus, *Dmrt1* was proven to play an important role in the characteristic and behavior maintenance of induced eSLCs.

**Fig. 6:**
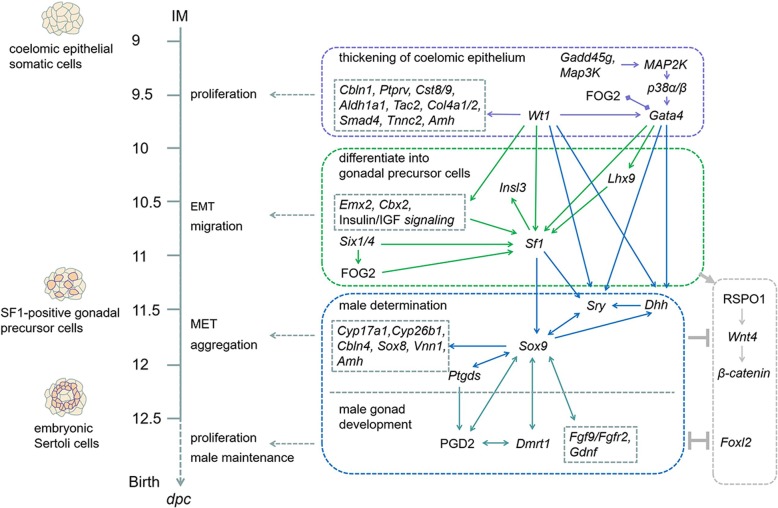
Molecular pathways map in derivation of eSCs. A speculated stepwise molecular pathway map in derivation process of mouse eSCs in reference to the conclusions made in this work and all the existing studies. The pathways map was divided into four phases. *Wt1* and *Gata4* played important roles since phase I in the thickening of coelomic epithelium and activated relevant downstream factors. In phase II, *Sf1* was activated by joint control of upstream factors and played a key role in differentiation of coelomic epithelial somatic cells to gonadal precursor cells including SF1-positive cells and SF1-positive gonadal precursor cells. In phase III, *Sox9* and *Sry* played key roles in initiation of the male determination, activation of relevant factors, and inhibiting female determining factors. In phase IV, *Dmrt1* and other relevant factors improved the male maintenance and facilitated the male gonad development

Based on the observed phenomena, molecular mechanism found in this differentiation model and all the existing studies, a molecular pathway map from IM to eSCs was mapped and divided into four phases (Fig. 6). Phase I is the thickening of coelomic epithelium, taking *Wt1* and *Gata4* as the core factors [7, 8, 11, 13, 14, 23, 71]. In this scenario, some factors played a role in proliferation and development of coelomic epithelium including *Cbln1*, *Ptprv*, *Cst8*/9, *Aldh1a1*, *Tac2*, *Col4a1*/2, *Smad4*, and *Tnnc2*. Phase II is the differentiation from coelomic epithelial somatic cells to gonadal precursor cells, taking *Sf1* as the core factor [16]. In this phase, *Wt1*, *Gata4*, *Emx2*, *Cbx2*, Insulin*/*IGF signaling, *Lhx9*, *Six1/4*, and FOG2 jointly activated the expression of *Sf1* [59, 72]. *Sf1* activated *Insl3* [20]. Under the influence of these factors, coelomic epithelial somatic cells undergo EMT and migration and develop into SGPCs. Phase III is the male determination of SGPCs, taking *Sry* and *Sox9* as the core factors [21, 63]. In male determination, *Wt1*, *Gata4*, *Sf1*, and *Dhh* jointly activated the onset of *Sry*. *Sry* activated *Sox9*. *Sox9* continued to activate functional factors including *Ptgds*, *Cyp17a1*, *Cyp26b1*, *Cbln4*, *Sox8*, *Vnn1*, and *Amh* and inhibit female-determining factors including R-spondin 1 (RSPO1), *Wnt4*, and *β-catenin* [64]. Under the influence of these factors, the SGPCs underwent MET and aggregation and developed into eSCs. Phase IV is the male gonad development stage [18]. In this phase, the expression of *Dmrt1* was essential for the normal development of male gonad. *Dmrt1* activated functional factors including *Ptgds*, *Fgf9*, *Fgfr2*, and *Gdnf* and inhibited female determining factors including *Wnt4* and *Foxl2* to facilitate the cell proliferation and male maintenance of eSCs. These factors facilitated the eSCs to form seminiferous tubules and develop into testicle.

Conclusively, this molecular pathways map from coelomic epithelial cells to eSCs illustrated a whole picture of molecular mechanism involved during the derivation of eSCs. However, this map simply provided a fundamental base to reveal the mechanisms involved in gonadogenesis and male determination. A lot of mechanism studies still needed to be investigated and more evidences are required for verification. Hopefully, this differentiation model and molecular pathways map would be very useful for future advancing developmental mechanism researches.

## Conclusions

A differentiation model from mESCs to eSCs was established through spatiotemporal control of *Wt1*, *Gata4*, *Sf1*, *Sox9*, and *Dmrt1*. Based on the results of qPCR, IF, and FCM, a map of molecular pathways was proposed. This inducing approach provides an in vitro platform for studying the derivation of Sertoli cells with the tested methods of manipulating multiple factors.

## Supplementary information

Additional file 1: **Table S1.** Applicated plasmids.

Additional file 2: **Table S2.** Primers for complete gene sequences of the target factors.

Additional file 3: **Table S3.** Primers for qRT-PCR.

Additional file 4: **Table S4.** Lentivirus producing and packaging system.

Additional file 5: **Table S5.** Antibodies for immunofluorescence.

Additional file 6: **Table S6.** Antibodies for development stage identification by flow cytometry.

Additional file 7: **Figure S1.** Five constructed plasmids. FUW-lightO-GAVPO is made to produce element GAVPO for lightOn expression system. These plasmids are made for lentiviral transduction. FUW-lightO-*Wt1* was constructed from FUW-TetOn-GFP which was applicated to express gene *Wt1*. The rest plasmids in this paper were done in the same manner.

Additional file 8: **Figure S2.** Determination of induced eSLCs. (A) Pebble-like colonies (PCs) were observed in group mES + MEF at 10 days and 15 days. (B) PCs were observed in group mES + Trans at 10 days and 15 days. (C) Some epithelial-like cells derived from ESCs were marked by AMH and FASL antibodies. AMH result was a merged image of IF and microscope photograph. FASL/DAPI result was a merged image of green and blue fluorescence photograph. (D) FASL was determined in group mES + MEF and mES + Trans under immunohistochemistry (ICC). FASL^+^ cells showed dark brown. (E) Tubular-like structure was observed in group mES + Trans. (F) Ring-like structure was observed in group mES + Trans. ESCs were transduced by 5 factors in group mES + Trans. mES + MEF was control group.

Additional file 9: **Figure S3.** Transplant induced eSLCs and mature Sertoli cells in seminiferous tubule (ST). (A) Mature Sertoli cells and (B) induced eSLCs were injected into ST. The transplanted cells were stained by PKH26 showing red fluorescence. (C) The transverse slice of ST was performed by ICC. SSCs showed DDX4^+^. (D) The longitudinal slice of ST was performed by ICC. Sperm showed PGP9.5^+^.

Additional file 10: **Figure S4.** Coculture of SSCs and induced eSLCs. FCM result indicated the ratio of (A) AMH^+^ and (B) SOX9^+^ cells of FASL^+^ cells sorted from mES + Trans group. FCM result indicated the ratio of (C) C-kit^+^ and (D) CD9^+^ cells in extracted SSCs. (E) The induced eSLCs sorted from group mES + Trans were FASL^+^. (F) The eSLCs and SSCs were co-cultured for a week. Some cells showed long and narrow cell nucleus. (G) Under ICC, SSCs showed DDX4^+^. eSLCs and sperm-like cells were stained by hematoxylin showing blue. (H) Under ICC, sperm-like cells showed PGP9.5^+^.

## Data Availability

All data generated or analyzed during this study are included in this published article and its supplementary information files.

## Abbreviations

ANOVA: Analysis of variance
BSA: Bovine serum albumin
DOX: Doxycycline
EMT: Epithelial-mesenchymal transformation
eSCs: Embryonic Sertoli cells
eSLCs: Embryonic Sertoli-like cells
IM: Intermediate mesoderm
KO: Knockout
LIF: Leukemia inhibitory factor
MEFs: Mouse emrbyonic fibroblasts
mESCs: Mouse embryonic stem cells
MET: Mesenchymal-epithelial transformation
NEAA: Non-essential amino acids
RA: Retiotic acid
SD: Standard deviation
SGPCs: SF1-positive gonadal precursor cells
SPCs: SF1-positive precursor cells
