# Supplementary material for: Mapping molecular pathways for embryonic Sertoli cells derivation based on differentiation model of mouse embryonic stem cells
Source: Stem Cell Res Ther. 2020 Feb 26;11:85. doi: 10.1186/s13287-020-01600-2 (PMC7045406; doi:10.1186/s13287-020-01600-2)

Table S7 Sequence of constructed plasmids

Construct Name： FUW-lightO-GAVPO

Original Vector： FUW

Resistance/Selection Marker: Ampicillin for E.coli and Bleomycin for mammalian cells

RE site： *Bam*HI, *Eco*RI

Target gene Name： lightOn element-GAVPO

Target gene sequence:

GAVPO 1..1527 bp

ATGAAGCTACTGTCTTCTATCGAACAAGCATGCGATATTTGCCGACTTAAAAAGCTCAAGTGCTCCAAAGAAAAACCGAAGTGCGC
CAAGTGTCTGAAGAACAACTGGGAGTGTCGCTACTCTCCCAAAACCAAAAGGTCTCCGCTGACTAGGGCACATCTGACAGAAGTGG
AATCAAGGCTAGAAAGACTGGAAAGATCCATCGCCACCAGATCTCATACGCTCTACGCTCCCGGCGGTTATGACATTATGGGCTAT
CTGATTCAGATTATGAAGAGGCCAAACCCCCAAGTAGAACTGGGACCTGTTGACACGTCAGTTGCTCTGATTCTGTGCGACCTGAA
GCAAAAAGACACGCCAATTGTGTACGCCTCGGAAGCTTTTCTCTATATGACAGGATACAGCAATGCGGAGGTCTTGGGGAGAAACT
GCCGTTTTCTTCAGTCACCCGACGGAATGGTCAAGCCGAAATCGACAAGGAAGTACGTCGACTCCAACACGATCAATACGATGAGG
AAAGCGATTGATAGGAACGCCGAGGTGCAGGTTGAGGTGGTCAATTTTAAGAAGAACGGCCAACGGTTTGTCAACTTCTTGACGAT
GATTCCGGTGCGAGATGAAACAGGGGAATACCGGTACAGCATGGGTTTCCAGTGCGAAACGGAACTGCAGTACCCATACGATGTTC
CAGATTACGCTGAATTCCAGTACCTGCCAGATACAGACGATCGTCACCGGATTGAGGAGAAACGTAAAAGGACATATGAGACCTTC
AAGAGCATCATGAAGAAGAGTCCTTTCAGCGGACCCACCGACCCCCGGCCTCCACCTCGACGCATTGCTGTGCCTTCCCGCAGCTC
AGCTTCTGTCCCCAAGCCAGCACCCCAGCCCTATCCCTTTACGTCATCCCTGAGCACCATCAACTATGATGAGTTTCCCACCATGG
TGTTTCCTTCTGGGCAGATCAGCCAGGCCTCGGCCTTGGCCCCGGCCCCTCCCCAAGTCCTGCCCCAGGCTCCAGCCCCTGCCCCT
GCTCCAGCCATGGTATCAGCTCTGGCCCAGGCCCCAGCCCCTGTCCCAGTCCTAGCCCCAGGCCCTCCTCAGGCTGTGGCCCCACC
TGCCCCCAAGCCCACCCAGGCTGGGGAAGGAACGCTGTCAGAGGCCCTGCTGCAGCTGCAGTTTGATGATGAAGACCTGGGGGCCT
TGCTTGGCAACAGCACAGACCCAGCTGTGTTCACAGACCTGGCATCCGTCGACAACTCCGAGTTTCAGCAGCTGCTGAACCAGGGC
ATACCTGTGGCCCCCCACACAACTGAGCCCATGCTGATGGAGTACCCTGAGGCTATAACTCGCCTAGTGACAGGGGCCCAGAGGCC
CCCCGACCCAGCTCCTGCTCCACTGGGGGCCCCGGGGCTCCCCAATGGCCTCCTTTCAGGAGATGAAGACTTCTCCTCCATTGCGG
ACATGGACTTCTCAGCCCTGCTGAGTCAGATCAGCTCCGACTACAAAGACGATGATGACAAGTAA

Vector Map:

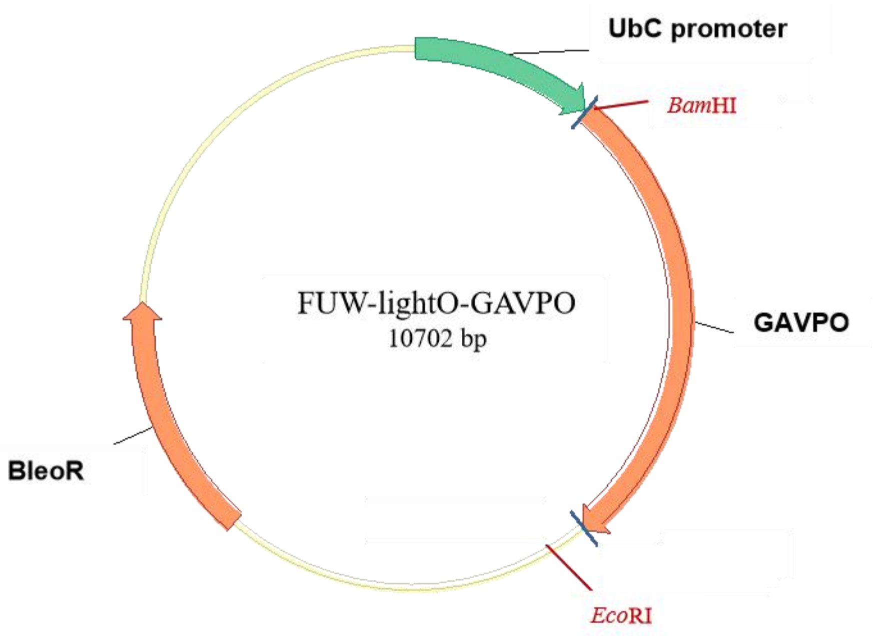
UbC promoter
BamHI
FUW-lightO-GAVPO
10702 bp
GAVPO
BleoR
EcoRI

Construct Name: FUW-lightO-*Wt1*

Original Vector: FUW-TetON-GFP

Resistance/Selection Marker: Ampicillin for E.coli and Bleomycin for mammalian cells

RE site: *Pst*I, *Bsr*GI

Target gene Name: lightOn element-*Wt1*

Target gene sequence:

CDS	*PolyA-5×UAS*$_G$*-Wt1-BGH polyA*	1..3694 bp
PolyA	bases 1..181 bp	
5×UAS$_G$	bases 182..300 bp	
Wt1	bases 378..3469 bp	
BGH polyA	bases 3470..3694 bp	

1 CTTGGAGCGG CCGCAATAAA ATATCTTTAT TTTCATTACA TCTGTGTGTT GGTTTTTTGT GTGAATCGAT

71 AGTACTAACA TACGCTCTCC ATCAAAACAA AACGAAACAA AACAAACTAG CAAAATAGGC TGTCCCCAGT

141 GCAAGTGCAG GTGCCAGAAC ATTTCTCTAT CGATAGGTAC CGAGTTTCTA GACGGAGTAC TGTCCTCCGA

211 GCGGAGTACT GTCCTCCGAC TCGAGCGGAG TACTGTCCTC CGATCGGAGT ACTGTCCTCC GCGAATTCCG

281 GAGTACTGTC CTCCGAAGAC GCTAGCGGGG GGCTATAAAA GGGGGTGGGG GCGTTCGTCC TCACTCTAGA

351 TCTGCGATCT AAGTAAGCTT GGCCACCTGT GTGAATGGAG CGGCCGAGCA TCCTGGCTCC TCCTCCTTCC

421 CTGCTGCCGG CCCCTCTTAT TTGAGCTTTG GGAAGCTGGG GGCAGCCAGG CAGCTGGGGT AAGGAGTTCA

491 AGGCAGCGCC CACACCCGGG GCTCTCCGCA ACCCGACCGC CTGCCTGCTC CCCCTTTCCT TTTCCCGCCC

561 TCCCTCCCAC CCACTCATTC ACCCACCCAC CCAGAGAGAG GACGGCAGCC CAGGAACCCG GGCCCGCCGC

631 CTCCTCGCCG CGATCCTGGA CTTCCTCCTG TCGCAGGAGC CGGCTTCCAC GTGTGTCCCG GAGCCGGCGT

701 CTCAGCACAC GCTCCGCCGG GAGCCCGGGT GCGTCCAGCA GCCGGAGCAA CCTGGGGACC GAGGCCCCCG

771 GAGCGCCTGG GCCAAGTCCA GCGCCGAGAA TCCGCAGGAT CGCAGGAGCG GAGAACCGTC CGCATCCGAG

841 CCGCACCTCA TGGGTTCCGA CGTGCGGGAC CTGAACGCGC TGCTGCCCGC TGTGTCTTCG CTGGGCGGCG

911 GCGGCGGCGG CTGCGGGCTC CCTGTGAGCG GCGCAGCGCA GTGGGCGCCC GTGTTGGACT TCGCGCCTCC

981 GGGCGCCTCG GCTTACGGGT CGCTGGGCGG TCCCGCGCCT CCTCCCGCTC CGCCGCCGCC TCCGCCGCCA

1051 CCCCACTCCT TCATCAAACA GGAGCCCAGC TGGGGCGGCG CCGAGCCACA CGAGGAGCAG TGCCTGAGCG

1121 CCTTCACCTT GCACTTCTCG GGCCAGTTCA CCGGTACAGC CGGGGCCTGT CGCTACGGAC CCTTCGGTCC

1191 TCCCCCGCCC AGCCAGGCGT CCTCGGGCCA GGCCAGGATG TTCCCCAATG CGCCCTACCT GCCCAGCTGC

1261 CTGGAGAGCC AGCCTACCAT CCGCAACCAA GGATACAGCA CGGTCACTTT CGACGGGGCG CCCAGCTATG

1331 GCCACACGCC CTCGCATCAC GCGGCGCAGT TCCCCAACCA TTCCTTCAAA CACGAGGACC CCATGGGCCA

1401 GCAGGGCTCG CTGGGCGAGC AGCAGTACTC CGTGCCACCT CCGGTGTATG GCTGCCACAC CCCTACTGAC

1471 AGTTGCACAG GCAGCCAGGC CCTGCTCCTG AGGACGCCCT ACAGCAGTGA CAATTTATAC CAAATGACCT

1541 CCCAGCTTGA ATGCATGACC TGGAATCAGA TGAACCTAGG AGCTACCTTA AAGGGAATGG CTGCTGGGAG

1611 CTCCAGCTCA GTGAAATGGA CAGAAGGGCA GAGCAACCAC GGCACAGGGT ATGAGAGTGA GAACCACACG

1681 GCCCCCATCC TCTGTGGTGC CCAGTACAGA ATACACACCC ACGGGGTCTT CCGAGGCATT CAGGATGTGC

1751 GGCGTGTATC TGGAGTGGCC CCAACTCTTG TCCGGTCAGC ATCTGAAACC AGTGAGAAAC GTCCTTTCAT

1821 GTGTGCATAC CCAGGCTGCA ATAAGAGATA TTTTAAGCTG TCCCACTTAC AGATGCATAG CCGGAAGCAC

1891 ACTGGTGAGA AACCATACCA GTGTGACTTC AAGGACTGCG AGAGAAGGTT TTCTCGCTCA GACCAGCTCA

1961 AAAGACACCA AAGGAGACAC ACAGGTGTGA AACCATTCCA GTGTAAAACT TGTCAGCGAA AGTTTTCCCG
2031 GTCCGACCAT CTGAAGACCC ACACCAGGAC TCATACAGGT AAAACAAGTG AAAAGCCCTT CAGCTGTCGG
2101 TGGCACAGTT GTCAGAAAAA GTTTGCGCGC TCAGACGAAT TGGTCCGCCA TCACAACATG CATCAGAGAA
2171 ACATGACCAA ACTCCAGCTG GCGCTTTGAG GGGTCCGACA CGGAGACAGT CCAGCATCCC AGGCAGGAAA
2241 GTGTGCAAAC TGCTTCCAAA TCTGATTTTG AAATTCCTCC CACTCACCTT TCAAAGGACA CGACTGTGGA
2311 TCTACATCCG ACTTCCAAGA CAGCACACCT GATTGACTGC ATCCTATCAG GTTTGCCGGA AGGAGTCGGT
2381 GCTCCGCCCA CTTTTGATTA ACTCACAGGC CTGAAAAAAG TGGTTCACGG TGTCTAGAAA GTCCATTGCT
2451 ATTGTCTGAA TTTTCTACTG TTAGAAGAAC CATTGTTGAT AATGCCCCCC GCCCCCCCCC CCGGGTTTCC
2521 TCTTCTCCTT TGTGATCATT TCCCCAGGAT TAGAGAGACT GTTACATTTT CTTTCATGGG ATATTTATAG
2591 GCCAGGGCAT GTGTATGTGC CTGCTAATGT AAACTCTGTC ATAGTTCCCA TTTACTAACT GCCCTAGAAA
2661 GAAATAAATC AGAGAGCAAG GCACCAGGGG CAAGAATCGT GCAGAATTTC AGAGGTCTGG CTGCAAACCT
2731 GGAAACCTGG AAGGCCAGAT GTAATTCTAC AGGCGATTGT TAAAGCTCAT AGGTTTTGAG TAACTGCATA
2801 GTAGGTTGGT ATTAACTAGA ACTCCTGTAT AGTTAGGACA GAGAGGAGCC TTCCTGCTCA GCTATTCACT
2871 CTGAACACTA GCACTGGGCT CTTAAGAAAT GATGTTTTAA GAGCAGAGAT CTTTTTTTAA TGTCTTTGAT
2941 TTATTTTTTA GTTGTAATTA GGTACATCCT CAGAGATGTA CTTTCCTCCT CTTGTGCAGG ATGTGGAGGA
3011 CTCAGTTCCA TCATCTGGGG CATCTTTAGA GTGTATAGAC CACACTGGTT ATGTGGCTTC AAGTTGTAAA
3081 AATTAAAATG ACTTTAAAAG AAACTAGGGG CTGGTCCAGG ATCTTCACTG GTAAGACTGT TCTTAAGTAA
3151 CTTAAGTATC TTTGAATCTG CAAGTATGTA GGGAAAAAAA AAAGATATAT TATTGTGAGG AAATCCATTG
3221 TTTAAAGGTG TGCGTGTGTT GTTGTTGTTT TTTAAAGGGA GGGAGTTTAT TATTTACTGT AGCTTGAAAT
3291 ACTGTGTAAA TATATATGTA TATATATGAT GTGCTCTTTG TCAACTAAAA TTAGGAGGTG TATGGTATTA
3361 GCTGCATCAC TGTGTGGATG TCAATCTTAC AGTGTATTGA TGATAATACT AAAAATGTAA CCTGCATCTT
3431 TTTCCACTTG GCTGTCAATT AAAGTCTATT CAAAAGGAAC TGTGCCTTCT AGTTGCCAGC CATCTGTTGT
3501 TTGCCCCTCC CCCGTGCCTT CCTTGACCCT GGAAGGTGCC ACTCCCACTG TCCTTTCCTA ATAAAATGAG
3571 GAAATTGCAT CGCATTGTCT GAGTAGGTGT CATTCTATTC TGGGGGGTGG GGTGGGGCAG GACAGCAAGG
3641 GGGAGGATTG GGAAGACAAT AGCAGGCATG CTGGGGATGC GGTGGGCTCT ATGG

Vector Map:

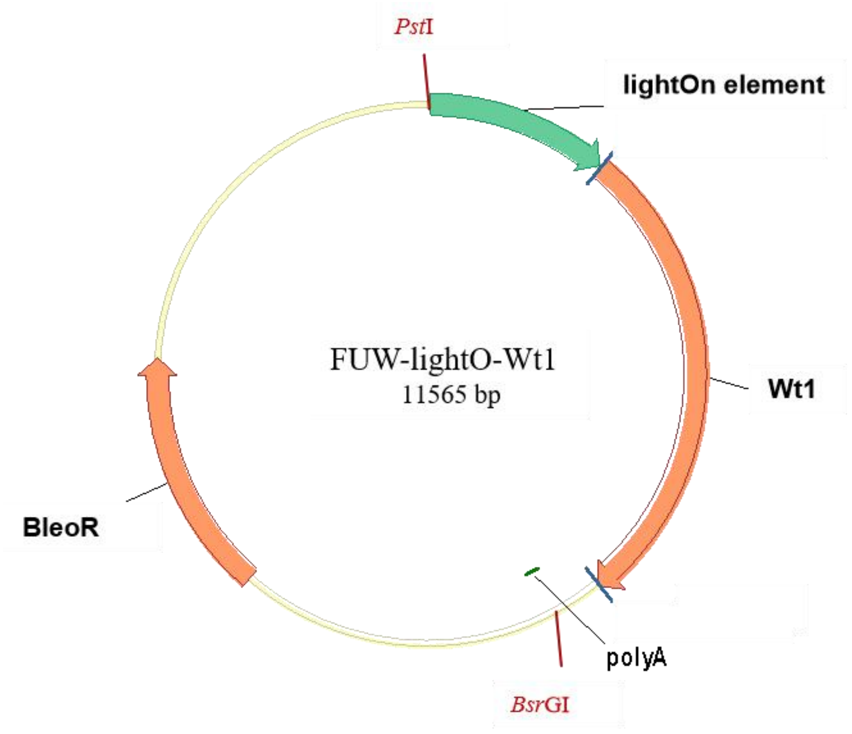
PstI
lightOn element
FUW-lightO-Wt1
11565 bp
Wt1
BleoR
polyA
BsrGI

Construct Name： FUW-lightO-*Gata4*

Original Vector： FUW-TetON-GFP

Resistance/Selection Marker: Ampicillin for E.coli and Bleomycin for mammalian cells

RE site： *Pst*I, *Bsr*GI

Target gene Name： lightOn element-*Gata4*

Target gene sequence:

CDS	*PolyA-5×UAS$_G$-Gata4-BGH polyA*	1..4010 bp
PolyA	bases 1..181 bp	
5×UAS$_G$	bases 182..300 bp	
Gata4	bases 378..3785 bp	
BGH polyA	bases 3786..4010 bp	

1 CTTGGAGCGG CCGCAATAAA ATATCTTTAT TTTCATTACA TCTGTGTGTT GGTTTTTTGT GTGAATCGAT

71 AGTACTAACA TACGCTCTCC ATCAAAACAA AACGAAACAA AACAAACTAG CAAAATAGGC TGTCCCCAGT

141 GCAAGTGCAG GTGCCAGAAC ATTTCTCTAT CGATAGGTAC CGAGTTTCTA GACGGAGTAC TGTCCTCCGA

211 GCGGAGTACT GTCCTCCGAC TCGAGCGGAG TACTGTCCTC CGATCGGAGT ACTGTCCTCC GCGAATTCCG

281 GAGTACTGTC CTCCGAAGAC GCTAGCGGGG GGCTATAAAA GGGGGTGGGG GCGTTCGTCC TCACTCTAGA

351 TCTGCGATCT AAGTAAGCTT GGCCACCGCG CCGGGAGCAG GGGACAAGCC GGAGGCCCGC AGAGTGGCCG

421 CCCGAGGCTC AGCCGCAGTT GCAGCTCCGC GGACTCACGG AGATCGCGCC GGTTTTCTGG GAAACTGGAG

491 CTGGCCAGGA CTGCCGCTTC GCTTCGAAGG GACCGGGCCC TCTTTGTCAT TCTTCGCTGG AGCCGCTCTG

561 GAGCTAGCAG CTGCGCCTGG GTGTGTAGCA GGCAGAAAGC AAGGACTAGG CTTCTTTAGC CGGTGGGTGA

631 TCCGAAGGCC TGCTCAGGGT GTTCGAGACC AGCCTGGACT GCGTCTGGGC ACCTCCAGCC TCTGGGCCCT

701 GGAATAGAGT CCGCCCTCCC GCACGATTTC TGGAGCAACC GCAAATCCAA TTTGGGATTT TCTTTTTCCT

771 GAGCAAACCA GAGCCTAGAG GTTTCTGCTT TGATGCTGGA TTTAATTCGT ATATATTTTG AGCGAGTTGG

841 GCCTCTCCTC GTTTTTTGAT CTCCGGTTGT TTTTTTTTTG GGGGGGGGGT TAGTTTTTGG GTTTTTGTTT

911 TGTTTTGTTT TGTTTTGATT TTTGGTGACA GTTCCGCACA CCCGCATTCT AGTTCTTGTC TGCCTCGTGC

981 TCAGAGCTTG GGGCGATGTA CCAAAGCCTG GCCATGGCCG CCAACCACGG CCCCCCGCCC GGCGCCTACG

1051 AAGCAGGTGG CCCTGGCGCC TTCATGCACA GCGCGGGCGC CGCGTCCTCG CCCGTCTACG TGCCCACTCC

1121 GCGGGTGCCG TCCTCTGTGC TGGGCCTGTC CTACCTGCAG GGCGGTGGCA GTGCCGCTGC AGCTGGAACC

1191 ACCTCGGGTG GCAGCTCCGG GGCCGGCCCG TCGGGTGCAG GGCCTGGGAC CCAGCAGGGT AGCCCTGGCT

1261 GGAGCCAAGC TGGAGCCGAG GGAGCCGCCT ACACCCCGCC GCCCGTGTCC CCGCGCTTCT CTTTCCCGGG

1331 GACTACTGGG TCCCTGGCGG CCGCTGCCGC CGCTGCCGCA GCCCGGGAAG CTGCAGCCTA CGGCAGTGGC

1401 GGCGGGGCGG CGGGCGCTGG TCTGGCTGGC CGAGAGCAGT ACGGGCGTCC GGGCTTCGCC GGCTCCTACT

1471 CCAGCCCCTA CCCAGCCTAC ATGGCCGACG TGGGAGCATC CTGGGCCGCA GCCGCTGCCG CCTCTGCCGG

1541 CCCCTTCGAC AGCCCAGTCC TGCACAGCCT GCCTGGACGG GCCAACCCTG GAAGACACCC CAATCTCGTA

1611 GATATGTTTG ATGACTTCTC AGAAGGCAGA GAGTGTGTCA ATTGTGGGGC CATGTCCACC CCACTCTGGA

1681 GGCGAGATGG GACGGGACAC TACCTGTGCA ATGCCTGTGG CCTCTATCAC AAGATGAACG GCATCAACCG

1751 GCCCCTCATT AAGCCTCAGC GCCGCCTGTC CGCTTCCCGC CGGGTAGGCC TCTCCTGTGC CAACTGCCAG

1821 ACTACCACCA CCACGCTGTG GCGTCGTAAT GCCGAGGGTG AGCCTGTATG TAATGCCTGC GGCCTCTACA

1891 TGAAGCTCCA TGGGGTTCCC AGGCCTCTTG CAATGCGGAA GGAGGGGATT CAAACCAGAA AACGGAAGCC

1961 CAAGAACCTG AATAAATCTA AGACGCCAGC AGGTCCTGCT GGTGAGACCC TCCCTCCCTC CAGTGGTGCC
2031 TCCAGCGGTA ACTCCAGCAA TGCCACTAGC AGCAGCAGCA GCAGTGAAGA GATGCGCCCC ATCAAGACAG
2101 AGCCCGGGCT GTCATCTCAC TATGGGCACA GCAGCTCCAT GTCCCAGACA TTCAGTACTG TGTCCGGCCA
2171 CGGGCCCTCC ATCCATCCAG TGCTGTCTGC TCTGAAGCTG TCCCCACAAG GCTATGCATC TCCTGTCACT
2241 CAGACATCGC AGGCCAGCTC CAAGCAGGAC TCTTGGAACA GCCTGGTCCT GGCTGACAGT CATGGGGACA
2311 TAATCACCGC GTAATCAGCG CCCCCCCTTC CCTCTTCAAA TTCCTGCTCG GACTTGGGAC GTGGGGGCCA
2381 GCAAAGTAAA AGGCTGGGGC ACCCTTGGCC AGCCCCTTTG TCTGGGAACA ACTCCTGAAG AACAACTGGG
2451 TAGAACTTGA AGTTGTTGAC AATCACTTAG GGATATGGGT GTTCCGGGTT GTTCAAACAC CTTTCCAGGT
2521 GGAGCACTGG AAAAGCCTGC GTTCTTACAG AGAAGCCCAC CTTGGCTGCA AGCACAGCAC AGTGAGGCAA
2591 GAGACTTCTT CCTTCCTTAT TCTCCACCTG CCTGTCCAGG ACAGACACAT AATCTCCTTC ACCCCAGCTC
2661 CCCACCCAGT TGTGGTGGTG GGTTTTTCTT TGTGATCCTA GAGTGGCTGT AGGGGCGGAG GCTTCAAGAC
2731 ACCATCTACA GTCTGAGCAG GGTGTCTACT TGTTGTAGAC TAGACATAGA AGCCCTGCCC TTGTCCAACA
2801 CTCCCCTTGC TTGAGGCATG GCACATCTCT GCATGTCCCA TACCAGATCT GACTCCAAAG TGCTGGGTTC
2871 AATGCAGATG TTACTGAATG CTTCCTGGGG AGATTAGGTG AGGGGAAGGC ACATCACCCA TCACACAGAA
2941 TAGCTTCATC AAATCGCAGC CTGGCCATGG TGCCTTCCCT TCCTCTCCCA GGAACATCAA ACCCCTTGCT
3011 CTCCAGCCTG AACATCTACC CTCTGCAAAA GTAGAGCCCA GTTGTGCAGC TAATGCCACT AGGTGCTATA
3081 TCCCAGCATC CTTTTCACCC CTTCACACAC AGGGGTTCCA AGGAGGAACA AAACCTGCTA CCAAAGCAGC
3151 CTTGGTGACT ATGGCTCATC TGCACCTCAG GGGGTGGGGG AGGGCCCTCT GGAGGTTGTG TCTACAGCAC
3221 AATACTGTTC CCAGGACTCT AGCTTGCTTG CCCCGAGCCT GCCAAGCCAA GCCCTCTTAA GTCAGACAGT
3291 TACCTGGCTC TGGGACTTTC TCCAGCACAG ATCCTTTGTC TAGAAAATAC AGACTGTTTG CAAAATAAAT
3361 TCAAAGCAGA AACAACTAAA GGAAATTTGT GAAAGGACAA AGGTGATAGA CGGGAGAAGA TGTCCCCAGG
3431 GCTGGCGGGA CAGTCATGAT AGCAGCTGTC CTAGGATTGG CCTCCCTCCC ATCTCCCACC ATTACTGGGG
3501 CTCCCAGAGA TTCTTCCTTG TCCTCATCAC CCACAGAGCT GTAGCCAACT GTGGCATTAC TTTATTTTAC
3571 CCAAAATTCC CAGCCCCACC CCTAAACCTT ACTGGCCGTA GCAGAGAATA GCTTCGAACC AAGATTCTGT
3641 TGTAATCATT TTCGCTGTTT CTCCCTCAAG GCCGCCTTCC CCATGCCTGC CCCTCCTCCA CAACCCGTTA
3711 ACATTGTCTT AAGGTGAAAT GGCTGTAAAA TCAGTATTTA ACTAATAAAT TTATCTGTAT TCCTGTTTCC
3781 TCCGACTGTG CCTTCTAGTT GCCAGCCATC TGTTGTTTGC CCCTCCCCCG TGCCTTCCTT GACCCTGGAA
3851 GGTGCCACTC CCACTGTCCT TTCCTAATAA AATGAGGAAA TTGCATCGCA TTGTCTGAGT AGGTGTCATT
3921 CTATTCTGGG GGGTGGGGTG GGGCAGGACA GCAAGGGGGA GGATTGGGAA GACAATAGCA GGCATGCTGG
3991 GGATGCGGTG GGCTCTATGG

Vector Map:

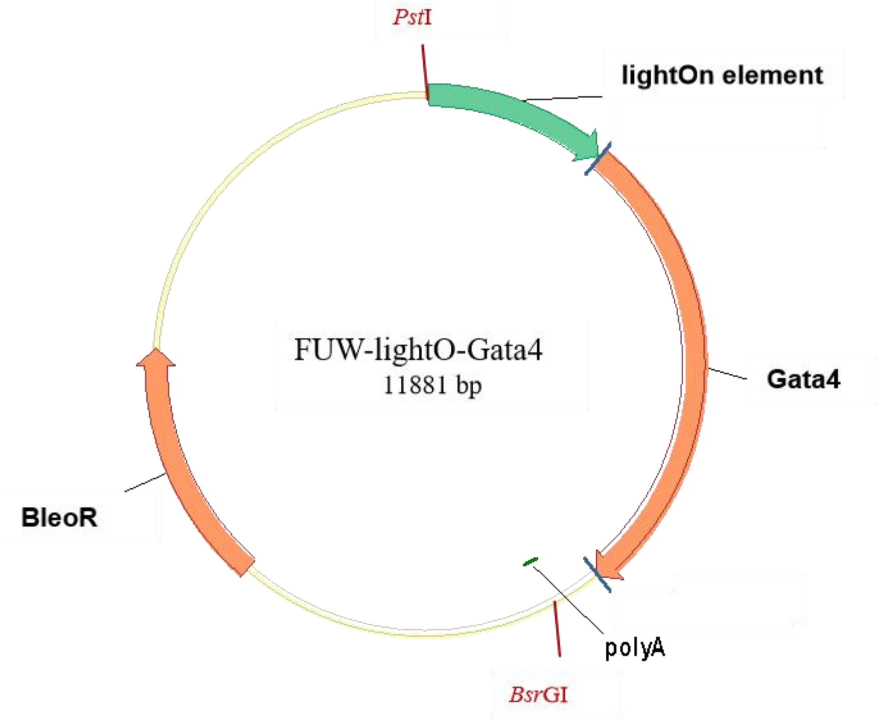

PstI
lightOn element
Gata4
FUW-lightO-Gata4
11881 bp
BleoR
polyA
BsrGI

Construct Name： FUW-lightO-*Sf1*

Original Vector： FUW-TetON-GFP

Resistance/Selection Marker: Ampicillin for E.coli and Bleomycin for mammalian cells

RE site： *Pst*I, *Bsr*GI

Target gene Name： lightOn element-*Sf1*

Target gene sequence:

CDS	*PolyA-5×UAS$_G$-Sf1-BGH polyA*	1..3596 bp
PolyA	bases 1..181 bp	
5×UAS$_G$	bases 182..300 bp	
Sf1	bases 378..3371 bp	
BGH polyA	bases 3372..3596 bp	

1 CTTGGAGCGG CCGCAATAAA ATATCTTTAT TTTCATTACA TCTGTGTGTT GGTTTTTTGT GTGAATCGAT

71 AGTACTAACA TACGCTCTCC ATCAAAACAA AACGAAACAA AACAAACTAG CAAAATAGGC TGTCCCCAGT

141 GCAAGTGCAG GTGCCAGAAC ATTTCTCTAT CGATAGGTAC CGAGTTTCTA GACGGAGTAC TGTCCTCCGA

211 GCGGAGTACT GTCCTCCGAC TCGAGCGGAG TACTGTCCTC CGATCGGAGT ACTGTCCTCC GCGAATTCCG

281 GAGTACTGTC CTCCGAAGAC GCTAGCGGGG GGCTATAAAA GGGGGTGGGG GCGTTCGTCC TCACTCTAGA

351 TCTGCGATCT AAGTAAGCTT GGCCACCGCA TATAGAGCGG CCACCCCGGC CCCGCGCGGC GGGTTCGCGA

421 AGCGCGCAGT GCGGCGGAGG GGCCCGGGTC CCAGGTGCGC CTCGAGCAGC CCCGCGGGCG GCGGGCGCGG

491 GGAGGCGGGG GCAGAGCGGG ACGGTCTCCC CGCCCTCGGC CCGAGTGGCC GTCATTGCGG GCGGGCGGCG

561 CTGAGCCCGG GTGCGGCTTC ATCGCGGGCG GCGGCGCGGC CAAAAGGCGG ACGCCGCGGG CATGGACTAT

631 TCGTACGACG AGGACCTGGA CGAGCTGTGT CCAGTGTGTG GTGACAAGGT GTCGGGCTAC CACTACGGGC

701 TGCTCACGTG CGAGAGCTGC AAGGGCTTCT TCAAGCGCAC AGTCCAGAAC AACAAGCATT ACACGTGCAC

771 CGAGAGTCAG AGCTGCAAAA TCGACAAGAC GCAGCGTAAG CGCTGTCCCT TCTGCCGCTT CCAGAAGTGC

841 CTGACGGTGG GCATGCGCCT GGAAGCTGTG CGTGCTGATC GAATGCGGGG TGGCCGGAAC AAGTTTGGGC

911 CCATGTACAA GAGAGACCGG GCCTTGAAGC AGCAGAAGAA AGCACAGATT CGGGCCAATG GCTTCAAGCT

981 GGAGACCGGA CCACCGATGG GGGTGCCCCC GCCACCCCCT CCCCCACCGG ACTACATGTT ACCCCCTAGC

1051 CTGCACGCAC CGGAGCCCAA GGCCCTGGTC TCTGGCCCAC CCAGTGGGCC GCTGGGTGAC TTTGGAGCCC

1121 CATCTCTACC CATGGCTGTG CCTGGTCCCC ACGGACCTCT GGCTGGCTAC CTCTATCCTG CCTTCTCTAA

1191 CCGCACCATC AAGTCTGAGT ATCCAGAGCC CTATGCCAGC CCCCCACAAC AGCCAGGGCC ACCCTACAGC

1261 TATCCAGAGC CCTTCTCAGG AGGGCCCAAT GTACCAGAGC TCATATTGCA GCTGCTGCAA CTAGAGCCAG

1331 AGGAGGACCA GGTGCGCGCT CGCATCGTGG GCTGTCTGCA GGAGCCAGCC AAAAGCCGCT CTGACCAGCC

1401 AGCGCCCTTC AGCCTCCTCT GCAGAATGGC CGACCAGACC TTTATCTCCA TTGTCGACTG GGCACGAAGG

1471 TGCATGGTCT TTAAGGAGCT GGAGGTGGCT GACCAGATGA CACTGCTGCA GAACTGTTGG AGCGAGCTGC

1541 TGGTGTTGGA CCACATCTAC CGCCAGGTCC AGTACGGCAA GGAAGACAGC ATCCTGCTGG TTACTGGACA

1611 GGAGGTGGAG CTGAGCACAG TGGCTGTGCA GGCTGGCTCC CTGCTGCACA GCCTGGTGCT GCGGGCCCAA

1681 GAGTTAGTGC TCCAGTTGCA TGCACTGCAG CTGGACCGCC AGGAGTTCGT CTGTCTCAAG TTCCTCATCC

1751 TCTTCAGCCT CGATGTGAAA TTCCTGAACA ACCACAGCCT CGTAAAGGAC GCCCAGGAAA AGGCCAACGC

1821 TGCCCTGTTG GATTACACCT TGTGTCACTA CCCACACTGC GGGGACAAAT TCCAGCAGTT GCTATTGTGC

1891 CTGGTGGAGG TGCGGGCCCT GAGCATGCAG GCCAAGGAGT ACCTGTACCA CAAGCATTTG GGCAACGAGA

1961 TGCCCCGCAA CAACCTTCTC ATTGAGATGC TGCAGGCCAA GCAGACTTGA GCCTGGGTGC CAGGCAGCGG
2031 GCAATAGGCA GGGATGCCAC TGCCTCCAAA AGACTCCTTG CATTAGGTGA TCCAGGAGCC CTGTCACTAA
2101 GCCCCTGCCC CTGAGCTCCA GAGCTGTGTG TTTGGGCAAG GATGGGCGGG GATTGGCCGG GGCAGGTTGC
2171 CTTTACTAGC CATTGGCCTG TGTCCGCCAC TTGGAGTGCC CCAAAGGGGG CTTCTAACCA TTCCTTCCTC
2241 CATCAGCCCC CAGCTTTTTT TCCTGGTATC TGAGGTCCCA GGAGGAGGCT CAGGATTCCC TGGTGGGTCT
2311 GGATGTCCCT TGGGTCAGAG GTCATCCTTT CCCTCTCTCC TGTTATCAGA GGCAAAGGAA GGTCTACAGG
2381 CATCAATGAG GGCAAAGGAG GGGGTCTCCA GACTCCACTG AAGCAGGAAG TCCACTGTTG TAAACTGAGT
2451 TTGCTAAATT GGGTCCCCAG AGGATACCAT GAGAGTGGGT AGGGCAAAAA GAGCCCTTTC CGCCCTCTAC
2521 CCATCTAATT CTGATCCTCT ACCTGTAGGA GGACTTTGGT GTGATCATCC TTCTCCCAGG GCCCGGCTAC
2591 CCAGGGAGGA GGAGTCTGGT GTAGCCAACA TTCCTGCCCT AACCCTGCCC ATCACCAGCT GGCTGGGCTG
2661 GTATTTATCT GCAAGGTTGA AGTCACTGGG ATTCTTTTCC TTTCACCTAG ATAGTCCTTG GAAAGTGTGT
2731 GAGAGAGAAG TGGGCAGGAG ACAGACTGGG GACTGAGCTG GGATATGGGG ACTAGCATCA AAGCTTTCTC
2801 CTGACATCTC TTTCCAAGAG TCGGGGTGGC ATCTGTACCC CACCTCACCC CCGAGAAGTG CTATTGCTTG
2871 CCCTCTGCCT CAGCCCCACT AGGGGAACAA CAGGAGGCCT GCTGGGGCTT AGAGTCCGTG CAGGTGGGGA
2941 TATGGGTAAA TCTAGGAGAA CTCACAGATC TTTATATGAG GACAGTGCTG AGGACTTTCT CATGGCTCCA
3011 TCCTTTTGGT CCCTCGCCAC TACCCTTGAA GCTGGCTTCA GTTCCCTGGC TGCTGCTTTG CCTCCTGAAA
3081 GCCACTCTGT AGGACCAAGC ACTCGGGGGA GAGGCCTAAG CCATCCTCTG TTCCAGACTG GACATCCACT
3151 GTCTTTCCTG CTTTCGCGTC AGATTTACAG CTTATGCTAG GCCCACCCAA CTGGACAAGG CTGTCTCCTG
3221 TCTTCTACTA CCCTGGCTCA GCCCCCACCT CTGCCCCTGA AATGCGTGCT CCCACCAAGG CCAGAGACCC
3291 ACAGCCCCAA GACAAGAAGT GCCCTTATAA ACCCCTGCAG CCCTGCAGCC CTGAAATAAA TTTTGCAATT
3361 AGTTTCCAGT ACTGTGCCTT CTAGTTGCCA GCCATCTGTT GTTTGCCCCT CCCCCGTGCC TTCCTTGACC
3431 CTGGAAGGTG CCACTCCCAC TGTCCTTTCC TAATAAAATG AGGAAATTGC ATCGCATTGT CTGAGTAGGT
3501 GTCATTCTAT TCTGGGGGGT GGGGTGGGGC AGGACAGCAA GGGGGAGGAT TGGGAAGACA ATAGCAGGCA
3571 TGCTGGGGAT GCGGTGGGCT CTATGG

Vector Map:

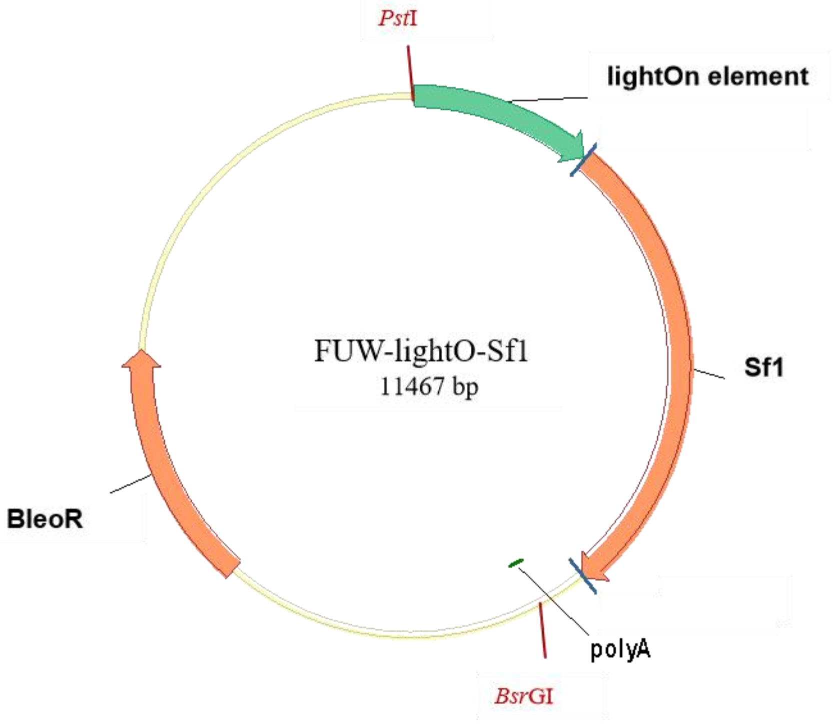

Construct Name： pLenti-CMV-*Dmrt1*

Original Vector： pLenti-CMV-GFP-Zeo

Resistance/Selection Marker: Ampicillin for E.coli and Bleomycin for mammalian cells

RE site： *Bam*HI, *Bsr*GI

Target gene Name： *Dmrt1*

Target gene sequence:

Dmrt1 1..2251 bp

1 AGAAAAAAGA AAAAAGAAAA GAGGTACAAC CCCCCACCCA CTCCCCGAGC TCTTCATTTA GACTACAGGT

71 CCAGCTGCGG TGCACCCGCC TCCTGCGCGC CTTCTAGGCA GCCGGCTGGG TGGATCCGGG AGCGGGAGAC

141 GAAGCTCCAT GCCGAACGAC GACACATTCG GCAAGCCCTC TACCCCGACG GAGGTTCCTC ACGCTCCCGG

211 GGCCCCGCCG CAGGGCAAAG CCGGAGGTTA CAGCAAAGCT GCCGGGGCGA TGGCTGGAGC GGCTGGAGGC

281 TCGGGCGCGG GGGGCAGCGG AGGTGCCTCG GGCTCCGGGC CGTCGGGCCT GGGTTCTGGA AGCAAGAAGT

351 CCCCGAGGCT GCCCAAATGC GCTCGCTGCA GGAACCACGG CTACGCGTCG CCGCTCAAGG GCCACAAGCG

421 CTTCTGCATG TGGCGGGATT GCCAGTGCAA GAAGTGCAGC CTGATTGCGG AGCGACAGCG GGTGATGGCC

491 GCGCAGGTGG CCCTGAGAAG ACAGCAGGCC CAGGAAGAAG AACTGGGTAT CAGCCACCCA ATTCCGCTGC

561 CCAGCGCAGC CGAGCTCCTG GTCAAAAGAG AGAATAATGC CAGCAACCCG TGCCTGATGG CCGAGAACAG

631 CAGCTCTGCC CAGCCCCCAC CGGCCAGCAC CCCAACCCCC GCTGCCTCAG AGGGACGCAT GGTCATCCAG

701 GATATTCCTG CTGTCACCAG CAGAGGGCAT ATGGAGAACA CATCTGACCT GGTATCAGAC CCCGCCTACT

771 ACAGCAGCTT TTACCAGCCT TCTCTGTTTC CTTACTACAA CAATCTGTAC AACTACCCCC AGTATTCCAT

841 GGCCTTGTCT GCTGAGTCCT CCTCAGGGGA GGTGGGGAAT TCCCTCGGGG GATCCCCCGT GAAGAACAGC

911 CTGCGGAGTC TCCCAGCACC TTACGTGCCT GCTCAGACTG GAAACCAGTG GCAGATGAAG ACCTCAGAGA

981 GCCGCCACCC AGTGAGCTCC CAGTACCGGA TGCACTCATA CTACGGGCCT CCCTCCTACC TGGGCCAGAG

1051 CATGTCCCAG ATCTTTACCT TCGAGGAAGG CCCCTCCTAC TCAGAAGCCA AAGCCAGTGT GTTCTCGCCC

1121 CCCAGCAGTC AAGATTCTGG CTTGGTCTCC CTCTCCAGCA GCTCTCCGAT GAGCAACGAG AGCTCGAAGG

1191 GAGTTCTGGA ATGTGAGTCT GCGTCCTCGG AACCTAGCAG CTACGCCGTC AACCAGGTCC TGGAAGAGGA

1261 TGAGGACGAG TGAGCCGCCG CCGGGGCTAC TCTGGTCACA GGGTGTTAGG CGGCTTGTTT TGTTATCGGC

1331 AGGGTTTGTT GTTATTTGAA TTGGCAGTGC TGGCTGCCCA TTCAGAAGTT TGGTTCATGT TAGAGTTAGG

1401 TTAAAACACT TGTAACAGTG TAGGATCCCA GACTACCATC TGCAAGAAGT AAGTGCCTGG CTCACCAAGT

1471 TTAAACAGTA ATGAGACTCG CTCCCTTGCC CAGAGCCTGT CCCTTTCCCT TCCTTCTCCA CTGTGTTGGT

1541 TTTTTTTTTT TGGGGGGGGG GGGGAGGATC TTCCAGAGAA AAGAATTGAC TGTATGCCCA AGTTACAATT

1611 ACATGCTACT TTTGGGGTTC AAATGAAATT TTCGGTGCCT TAGGTTGTAA CCAAGTTTTC AGGATTTTTA

1681 AGAAAACCAA GAAAATATAA ATTTAGCTCT AAGTACTTGA CCAGTGAGAA GAGCGGGCAA ACCTCTTTTT

1751 AGAATTCTGC AGACAGTAAA TGGAGACACT GTTTATGTTT TGGTGATGTG TTGGTGGCTG CACCCCACAC

1821 CCCAAATCCA AATGTAACAG GAATACAGTA AGAAGAAACC ACAGCGAGAG GAAACAGGGA AAACCTCCCC

1891 AAGCCTTACT TCTCAGATAA AGGGTAACTT AGAAAACAAT ACACCGTGAA GTGACCCCAG GAGGCATTTC

1961 CTGGGTGTTG TTCCTAGAAT GAGTTTAACT AACGCATACA TTTTCTTTAC CACAGGGGGA AAAATGGAGG

2031 CAATCTAGAC GATTTTGTAA CCTTAGGTTG TAATCTGGCT TGAAAATTAG TACATCTTTA AAAGTTACCA

2101 CTGATATTTG AGTTCATTAT TTTGTTAAAA AAATTGCTTT TAAAGTACTT ATGTTACAGG AAGCCATCCT

2171 GAAATGGAAC CGGTCTAAAA AAAAATAATT GTGGTGTGTG GTTGCAGCTG TACCTGAAAT AAAAACGTTA

2241 TTGATGACTG C

Vector Map:

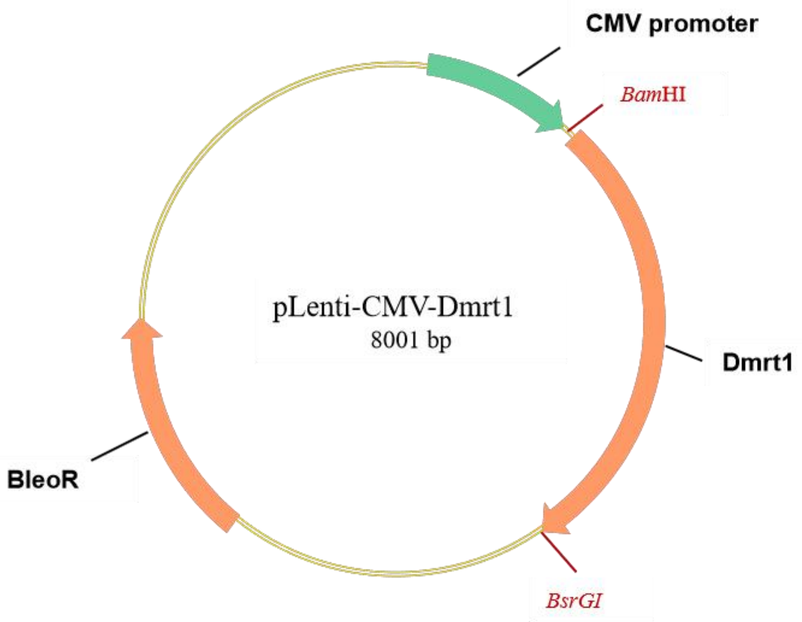

Supplement: Supplementary file 7 — Figure S1. Five constructed plasmids. FUW-lightO-GAVPO is made to produce element GAVPO for lightOn expression system. These plasmids are made for lentiviral transduction. FUW-lightO-Wt1 was constructed from FUW-TetOn-GFP which was applicated to express gene Wt1. The rest plasmids in this paper were done in the same manner. [file 13287_2020_1600_MOESM7_ESM.pdf]
