# Supplementary figures and images for: Mapping molecular pathways for embryonic Sertoli cells derivation based on differentiation model of mouse embryonic stem cells
Source: Stem Cell Res Ther. 2020 Feb 26;11:85. doi: 10.1186/s13287-020-01600-2 (PMC7045406; doi:10.1186/s13287-020-01600-2)

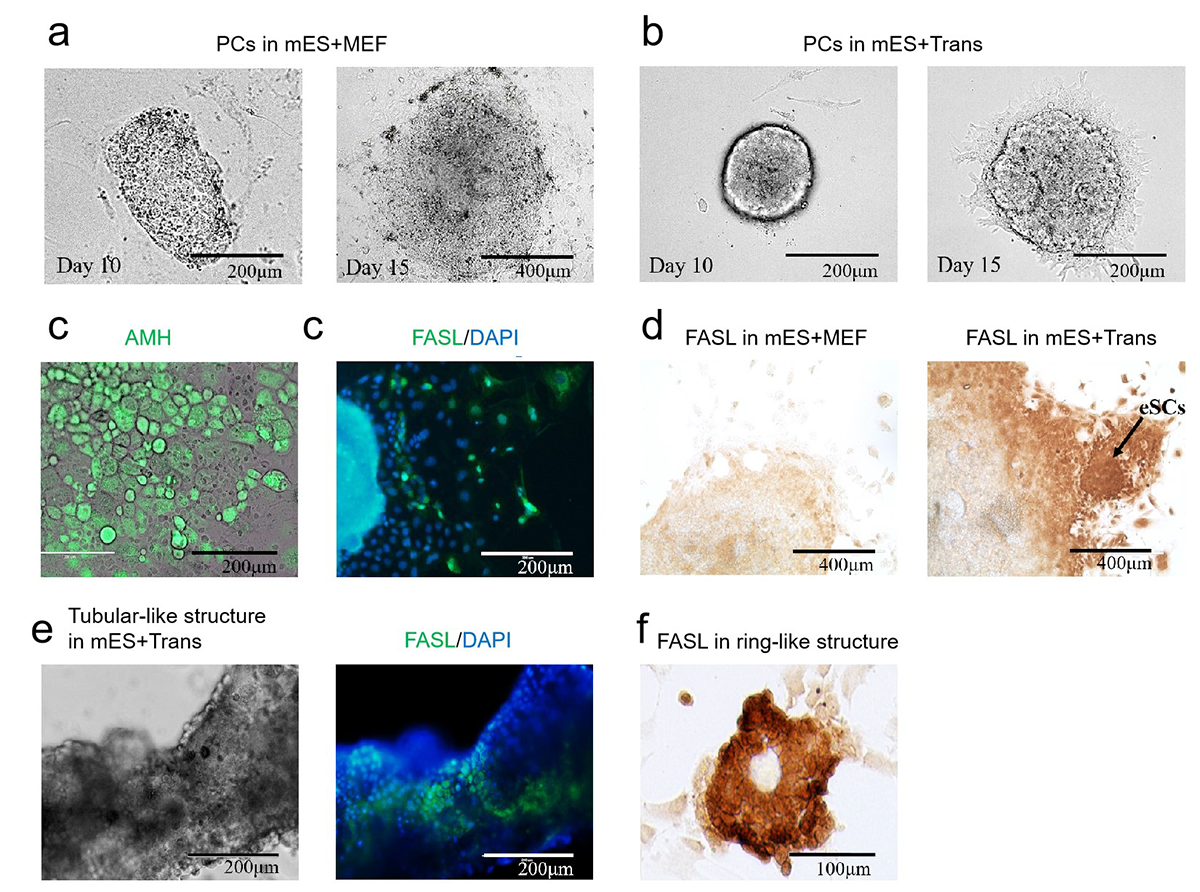

Supplement: Supplementary file 8 — Figure S2. Determination of induced eSLCs. (A) Pebble-like colonies (PCs) were observed in group mES + MEF at 10 days and 15 days. (B) PCs were observed in group mES + Trans at 10 days and 15 days. (C) Some epithelial-like cells derived from ESCs were marked by AMH and FASL antibodies. AMH result was a merged image of IF and microscope photograph. FASL/DAPI result was a merged image of green and blue fluorescence photograph. (D) FASL was determined in group mES + MEF and mES + Trans under immunohistochemistry (ICC). FASL+ cells showed dark brown. (E) Tubular-like structure was observed in group mES + Trans. (F) Ring-like structure was observed in group mES + Trans. ESCs were transduced by 5 factors in group mES + Trans. mES + MEF was control group. [file 13287_2020_1600_MOESM8_ESM.tif]

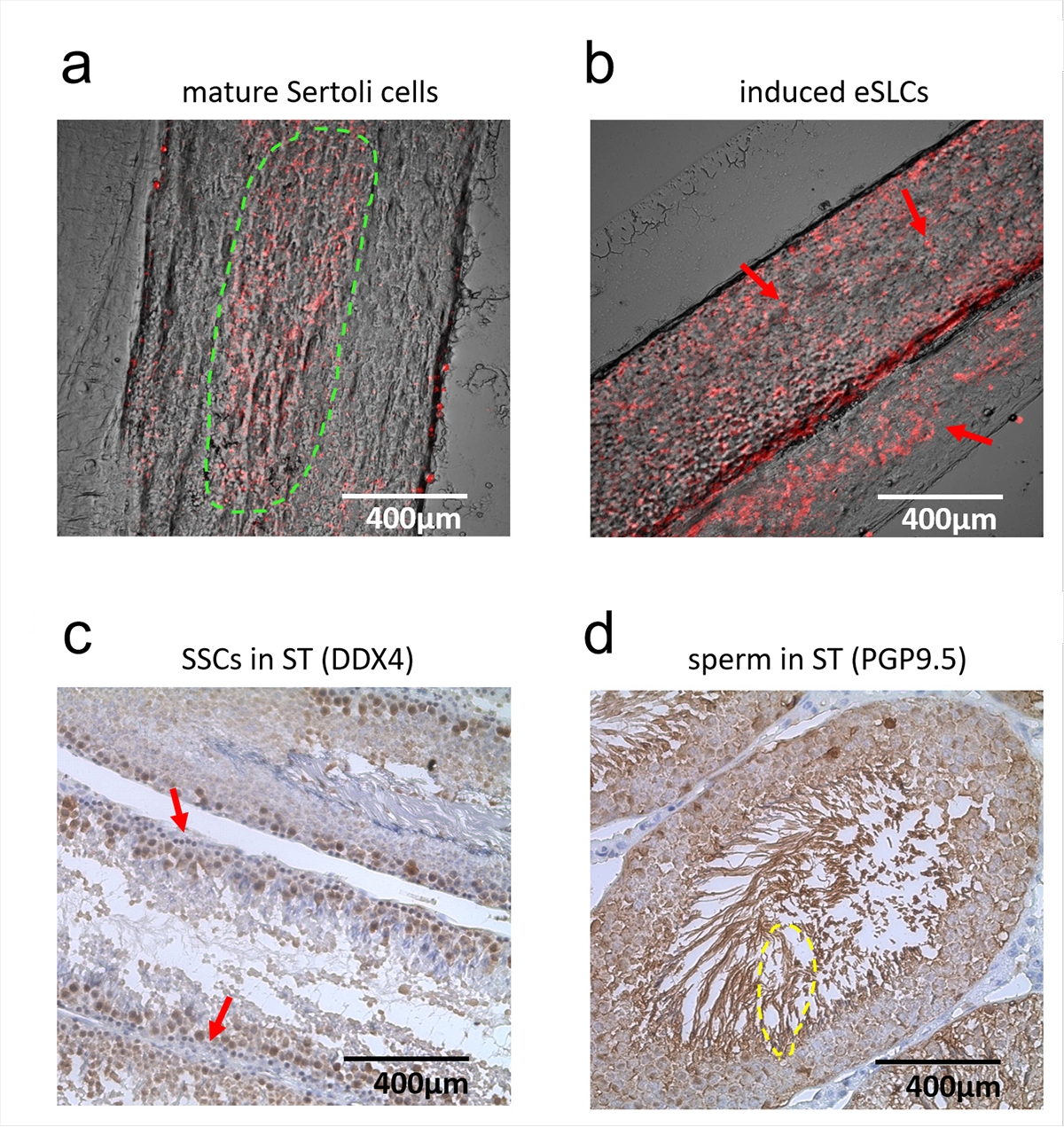

Supplement: Supplementary file 9 — Figure S3. Transplant induced eSLCs and mature Sertoli cells in seminiferous tubule (ST). (A) Mature Sertoli cells and (B) induced eSLCs were injected into ST. The transplanted cells were stained by PKH26 showing red fluorescence. (C) The transverse slice of ST was performed by ICC. SSCs showed DDX4+. (D) The longitudinal slice of ST was performed by ICC. Sperm showed PGP9.5+. [file 13287_2020_1600_MOESM9_ESM.tif]

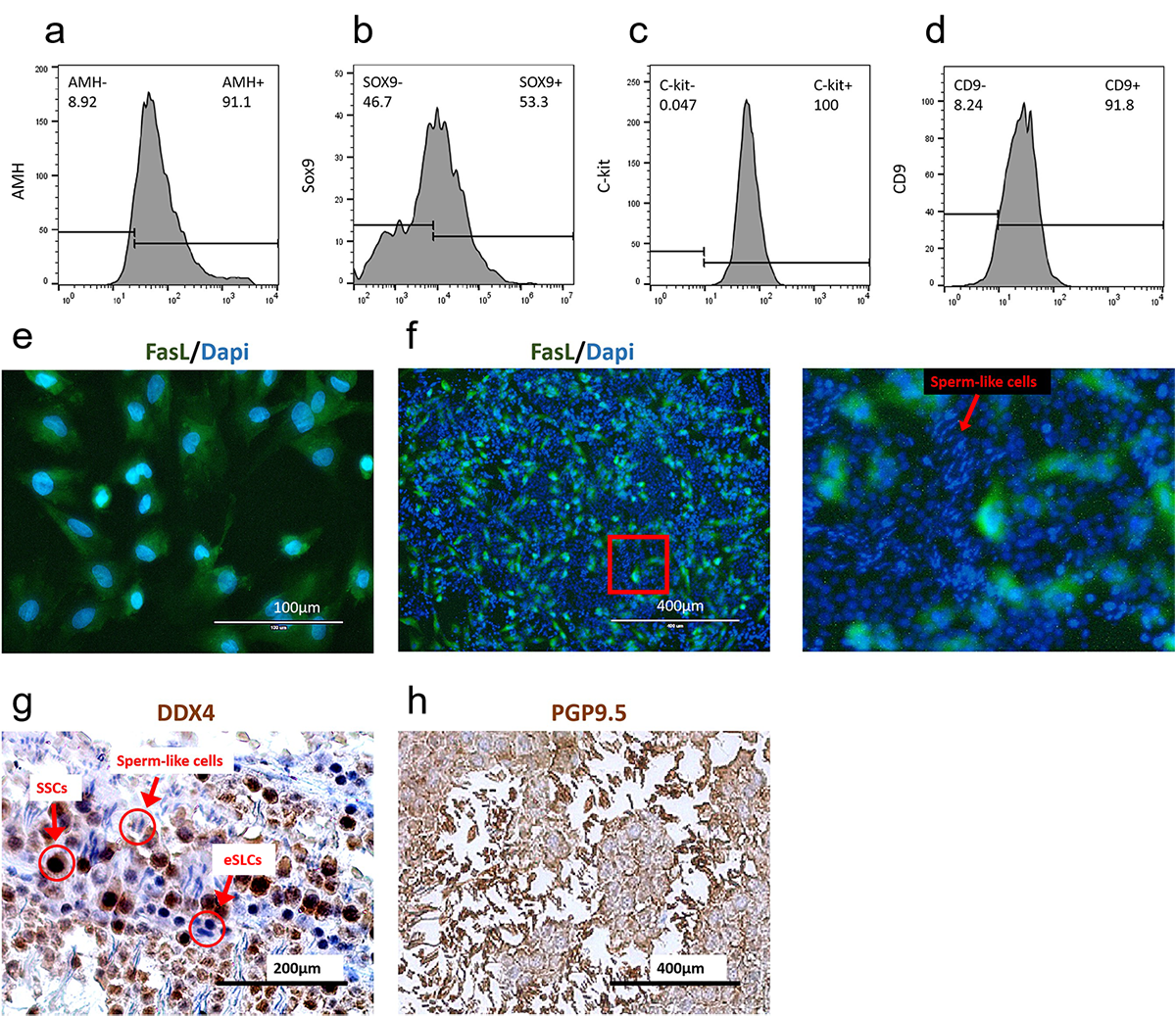

Supplement: Supplementary file 10 — Figure S4. Coculture of SSCs and induced eSLCs. FCM result indicated the ratio of (A) AMH+ and (B) SOX9+ cells of FASL+ cells sorted from mES + Trans group. FCM result indicated the ratio of (C) C-kit+ and (D) CD9+ cells in extracted SSCs. (E) The induced eSLCs sorted from group mES + Trans were FASL+. (F) The eSLCs and SSCs were co-cultured for a week. Some cells showed long and narrow cell nucleus. (G) Under ICC, SSCs showed DDX4+. eSLCs and sperm-like cells were stained by hematoxylin showing blue. (H) Under ICC, sperm-like cells showed PGP9.5+. [file 13287_2020_1600_MOESM10_ESM.tif]
